# *RHBDF2* gene functions are correlated to facilitated renal clear cell carcinoma progression

**DOI:** 10.1186/s12935-021-02277-0

**Published:** 2021-11-04

**Authors:** Lei Wang, Xiu-Xiu Liu, Yu-Meng Yang, Yan Wang, Yuan-Yuan Song, Shan Gao, Lu-Yuan Li, Zhi-Song Zhang

**Affiliations:** State Key Laboratory of Medicinal Chemical Biology, College of Pharmacy, Tianjin Key Laboratory of Molecular Drug Research, Nankai University, 38 Tongyan Road, Jinnan District, Tianjin, 300350 China

**Keywords:** Kidney renal clear cell carcinoma, Rhomboids, RHBDF2, Cancer prognosis, PD-L1

## Abstract

**Background:**

The rhomboids are a family of multi-transmembrane proteins, many of which have been implicated in facilitating tumor progression. Little is yet known, however, about rhomboid-associated biomarkers in cancers. An analysis of such biomarkers could yield important insights into the role of the rhomboids in cancer pathology.

**Methods:**

In this study, we carried out the univariate Cox regression analysis and compared gene expression patterns of several rhomboid genes in 30 types of cancers by using The Cancer Genome Atlas (TCGA) database and the methods delineated in Gene Expression Profiling Interactive Analysis (GEPIA). We then used datasets GSE47032, GSE126964, GSE68417 and 75 paired pathological specimens to verify the influences of the rhomboid genes in cancer progression. Moreover, we carried out Weighted Gene Correlation Network Analysis (WGCNA) to investigate gene-related functions and we exploited potential correlations between rhomboid genes expression and immune cell infiltration in cancer tissues. Furthermore, we constructed gene-knockdown cancer cell lines to investigate rhomboid gene functions.

**Results:**

We find that kidney renal clear cell carcinoma (KIRC) disease progression is affected by fluctuations in the expression of a number of the rhomboid family of genes and, more specifically, high levels of *RHBDF2* gene expression are a good indicator of poor prognosis of the disease, as patients with high RHBDF2 expression levels exhibit less favorable survival rates compared to those with low RHBDF2 levels. Silencing of the *RHBDF2* gene in KIRC cell lines leads to significantly diminished cell proliferation and migration; this is in good agreement with the identification of an enhanced presence of a number of cell growth and migration promoting signaling molecules in KIRC tumors. We found that, although high level of RHBDF2 correlated with increased infiltration of lymphocytes in cancer tissues, artificially overexpressed RHBDF2 led to an inhibition of the activity of the infiltrated immune cells through sustaining PD-L1 protein level. Furthermore, we show that RHBDF2 related cell migration and PD-L1 regulation were potentially mediated by EGFR signaling pathway.

**Conclusions:**

*RHBDF2* gene functions are correlated to facilitated renal clear cell carcinoma progression and may serve as a critical prognostic biomarker for the disease.

**Supplementary Information:**

The online version contains supplementary material available at 10.1186/s12935-021-02277-0.

## Background

Members of the rhomboid superfamily are six- or seven-transmembrane proteins, which have been shown to be widely present and may take part in a variety of important biological processes, including cytokine secretion [1–5], protein quality control [6–9], epithelial cell polarity [10], subcellular transport [11], and mitochondrial function regulation [12]. They are also shown to be associated with disease development such as in cancers [13–17] and autoimmune diseases [18–20]. Rhomboid proteins may be categorized in two groups, namely the proteolytically active or inactive [21]. The proteolytically active rhomboids include RHBDD1, RHBDL1, RHBDL2, RHBDL3 and PARL [22], that are capable of catalyzing the cleavage of their substrates and regulating related pathways. For example, they cleavage the pro-ligands of epidermal growth factor receptor (EGFR) and activate the EGFR signaling pathway [4, 16, 23]. The proteolytically inactive group includes RHBDF1, RHBDF2 (also known as iRhom1 and iRhom2, respectively) [24] were also able to activate the EGFR pathway through activation of EGFR ligands. RHBDF1 participates in GPCR-mediated transactivation of EGFR growth signals and RHBDF2 drives EGFR activation through an enhancement of the secretion of EGFR ligands [5, 13, 14]. The role of rhomboids in cancer progression is an important but under-explored subject in cancer research. We therefore set out to explore the gene expression patterns of rhomboids in a variety of cancers and their relationships to cancer progression, with a specific attention to associated biomarkers.

Kidney cancers account for approximately 2% of adult malignancies and is among the most prevalent cancers worldwide [25]. About 80% of kidney cancer cases are renal clear cell carcinoma (KIRC) [26]. In addition, symptoms of KIRC are often insidious in the early stage, which could explain why many patients are diagnosed when the disease is already in the advanced stages [27, 28]. The five-year overall survival (OS) rate of KIRC patients at early stage is up to 90%, whereas the disease becomes almost incurable in advanced stages[29]. Chemotherapy or partial resection are the main methods of treatment, yet local recurrence or distant metastasis often occurs [30]. In recent years, immune checkpoint inhibitors (ICI) which can block the PD-1/PD-L1 or CTLA-4 T cell inhibitory receptor, have been shown to be encouragingly effective in advanced renal-cell carcinoma [31, 32]. But some effects of immunotherapy are not durable. Discovery of new biomarker for disease progression or treatment option will undoubtedly facilitate the diagnosis and treatment of the disease [33].

In this study we determined the gene expression patterns of rhomboids in 30 types of cancers by using The Cancer Genome Atlas (TCGA) and the Gene Expression Profiling Interactive Analysis (GEPIA) databases. We then focused on RHBDF2 expression in KIRC and analyzed lymphocyte infiltration, immune checkpoints, and their relationships to disease progression. Our findings indicate that *RHBDF2* gene functions significantly contribute to the immunosuppressive microenvironment in KIRC. These data are consistent with the view that increased RHBDF2 may serve as a critical biomarker of poor prognosis of renal clear cell carcinoma as well as a potential therapeutic target.

## Methods

### Data source

Gene expression data of 30 types of cancers in TCGA database (https://portal.gdc.cancer.gov/) was downloaded from GDC API, and all sequencing data was normalized to TPM. Essential pathologic information and survival data of each individual cancer case were from TCGA database and TCGA Pan-Cancer Clinical Data Resource [34]. Gene expression datasets, GSE68417, GSE126964, GSE47032 and GSE167093, were downloaded from Gene Expression Omnibus (GEO) databases (https://www.ncbi.nlm.nih.gov/gds/). The GSE68417 [35] series on the platform GPL6422 (Affymetrix Human Gene 1.0 ST Array) includes 14 normal samples, 6 tumor benign samples, 13 low grade samples and 16 high grade samples of KIRC. The GSE126964 [36] series on the platform GPL20795 (HiSeq X Ten) contains 55 tumor samples and 11 matched normal samples from Chinese KIRC patients. The GSE47032 [37] series on the platform GPL5175 (Affymetrix Human Exon 1.0 ST Array) contains 10 KIRC tumor samples and their matched non-tumor samples. The GSE167093 on the platform GPL10558 (Illumina HumanHT-12 V4.0 expression beadchip) contains 609 renal tumors and 256 non-tumor renal tissues [38].

### Gene expression analysis in GEPIA

Rhomboid genes expression difference between tumor and normal tissues of adrenocortical carcinoma (ACC), KIRC and brain lower grade glioma (LGG) was analyzed by GEPIA [39] with the datasets in TCGA and The Genotype-Tissue Expression projects (GTEx). GEPIA, a web-based tool, (http://gepia.cancer-pku.cn/index.html) provides multiple interactive functions including differential expression analysis.

### Immune infiltration related analysis

We first analyzed the infiltration of immune cells in KIRC and GSE68417 with the ESTIMATE algorithm [40] (https://bioinformatics.mdanderson.org/estimate/rpackage.html). Then we calculated the Pearson correlation coefficient of immune infiltration scores and RHBDF2 expression and drew the scatter plot with “ggstatsplot” R package (https://cran.r-project.org/web/packages/ggstatsplot/index.html). The infiltrating immune cell types analysis was performed by TIMER2.0 (http://timer.cistrome.org/) which is an online analysis tool for scoring immune infiltrates across diverse cancer types by multiple immune deconvolution methods, including TIMER, CIBERSORT, quanTIseq, xCell, MCP-counter and EPIC algorithm [41–43]. Gene expression data in KIRC-TCGA was analyzed by TIMER algorithm, and patients were grouped according to the median of CD8^+^ T cell infiltration scores or macrophage infiltration scores for further survival analysis. Survival curve drawing was completed by R package, “survminer” (https://cran.r-project.org/web/packages/survminer/index.html).

### Functional enrichment analysis

Weighted Gene Correlation Network Analysis (WGCNA) was performed with R package “WGCNA” (https://cran.r-project.org/web/packages/WGCNA/index.html) to identify significant functional modules based on RHBDF2 expression in KIRC. The appropriate soft threshold and co-expression modules were obtained first, and then we acquired the Pearson correlation coefficient between each module and RHBDF2 expression. Three modules related to RHBDF2 expression were determined at last. The annotation of gene functions and gene-interactive networks in the RHBDF2-related modules were carried out by Network Analyst [44–48] (https://www.networkanalyst.ca/) and GeneMANIA [49] (http://genemania.org). The functional enriched pathways, depicted in Sankey diagram, was completed by R package “ggalluvial” [50]. Gene Set Enrichment Analysis (GSEA) was also performed by grouping the TCGA-KIRC with the median of RHBDF2, which was accomplished through locally downloaded GSEA software (https://www.gsea-msigdb.org) [51, 52].

### Materials

The human KIRC cell lines, 786-O and 769-P, were purchased from Cell Bank of the Chinese Academy of Sciences (Shanghai, China). The cells were cultured in RPMI1640 medium supplemented with 10% FBS. The pathological specimens of human renal clear cell carcinoma, including 75 paired tumor and adjacent tissues, were purchased from Shanghai Outdo Biotech Co., Ltd. RHBDF2 antibody (Proteintech, #23181-1-AP) was used for immunohistochemical staining and Western blotting. PD-L1 antibody (Boster, #BA1683-2), EGFR antibody (BBI, #D160292), phospho-EGFR antibody (Boster, #BM-4676) were used for Western blotting. BeyoClick™ EdU Cell Proliferation Kit with Alexa Fluor 488 (Beyotime, C0071S) was used to detect cell proliferation according to the method described in the product manual. Cell total RNA extraction was accomplished by EasrepTM total RNA extraction kit (Progmega, LS1040) and Hifair^®^ III 1st Strand cDNA Synthesis SuperMix (Yeasen, 11120ES60) was used for reverse transcription. UltraSYBR mixture (CWBIO, CW0957M) was use to real-time quantitative PCR. Matrigel (Corning #354248) was used to build the subcutaneous graft model. Gefitinib (Topscience #T1181) was used to block EGFR activation.

### Real-time quantitative PCR (RT-qPCR)

Hub genes of the WGCNA red module and immune checkpoints were detected by RT-qPCR. Total RNA extraction from the 786-O and 769-P cells were accomplished according to the manual of RNA extraction kit. Then we took 1μg RNA for cDNA synthesized and targeted genes detection. The thermal cycle program was as follows: denaturing for 15 s at 95 ℃, annealing and extension for 30 s at 60 ℃. Relative expression of the targeted genes was normalized to the ACTB by calculating the delta-Ct. Primers for RT-qPCR were as follows:

ITGB1-F: CAAGAGAGCTGAAGACTATCCCA,

ITGB1-R: TGAAGTCCGAAGTAATCCTCCT,

MAPK3-F: ATGTCATCGGCATCCGAGAC,

MAPK3-R: GGATCTGGTAGAGGAAGTAGCA,

PTK2-F: TGGTGCAATGGAGCGAGTATT,

PTK2-R: CAGTGAACCTCCTCTGACCG,

CD273-F: ACCGTGAAAGAGCCACTTTG

CD273-R: GCGACCCCATAGATGATTATGC

CD276-F: GTCCCTGAGTCCCAGAGTCG

CD276-R: ACGCAGCATCTTCCTGTGAG

LGALS9-F: TCTGGGACTATTCAAGGAGGTC

LGALS9-R: CCATCTTCAAACCGAGGGTTG

ACTB-F: CATGTACGTTGCTATCCAGGC,

ACTB-R: CTCCTTAATGTCACGCACGAT,

### *RHBDF2* knocked-down cell lines construction

Two independent shRNAs against RHBDF2 mRNA were synthesized by Genweiz, the sequences of which were (forward):

#1: GTGAAGCACTTTGCCTTTGATCTCGAGATCAAAGGCAAAGTGCTTCAC

#2: CACGGCTATTTCCATGAGGAACTCGAGTTCCTCATGGAAATAGCCGTG.

RHBDF2-shRNA sequences were constructed into pLKO.1 plasmid, and then transfected into 293T cells at a ratio of pLKO.1: psPAX2: pMD2.G = 4:2:1. The supernatant of culture medium was collected after 48 hours and filtered by a 0.45 μm filter to obtain the virus crude solution. The virus solution was used to infect 786-O cells and 769-P cells with the addition of 10 μg/mL of Polybrene. The control group of 786-O and 769-P were infected with empty vector virus. Cell culture media was changed after 24 hours, and puromycin was used to screen the infected cells with a 2 μg/mL concentration. Three days later, 786-O cells and 769-P cells with RHBDF2 knocked-down were obtained.

### Scratch healing assay

1×10^5^ tumor cells were seeded in 12-well plate and cultured with 10% FBS-RPMI1640 medium under 37℃ and 5% CO_2_ overnight. Then we draw a straight line in the middle of each well to create the wound and washed every well twice with PBS and change the medium to 2% FBS-RPMI1640. The microscopic images were taken by Nikon ECLIPSE Ts2 after 12 and 24 hours.

### Trans-well assay

3.5×10^4^ cells were seeded into the upper chamber suspending in 2% FBS RPMI1640 medium, while in the lower chamber the concentration of FBS was 20%. After cultured for 24 hours, cells were fixed with 4% paraformaldehyde for 15 mins at room temperature. Cells at the upper side were then erased and those at the bottom side were stained by 0.1% crystal violet solution. After washed with PBS, the bottom of the chamber was photographed and analyzed.

### Mouse model

We used 786-O cells and five-week-old nude mice to build the subcutaneous xenograft model. 786-O cells infected with empty virus or RHBDF2-shRNA virus were cultured in vitro and seeded into the abdominal subcutaneous tissue of mice. We mixed Matrigel and RPMI1640 medium at a 1:1 ratio, and then used the mixed medium to inject 1×10^7 ^cells per mouse.

### Statistical analysis

For the computerized analyses, we used the data of TCGA to carry out the univariate Cox regression and survival analyses, and the log-rank test was used for the significance test. WGCNA and GSEA were performed for RHBDF2 related functions and signaling pathways. Correlations between RHBDF2 expression and other factors mentioned in the study were mainly determined by the Pearson correlation coefficient. All the experiments in vitro in the study have been repeated at least 3 times. Data were subjected to student t-test, one-way ANOVA or two-way ANOVA for the statistical significance analyses. *P*-values of the differences smaller than 0.05 were of significance. For detail, the statistical analyses of each experiment were depicted in the relevant figure legends.

## Results

### Assessment of the prognostic value of rhomboid proteins in cancers

**Fig. 1:**
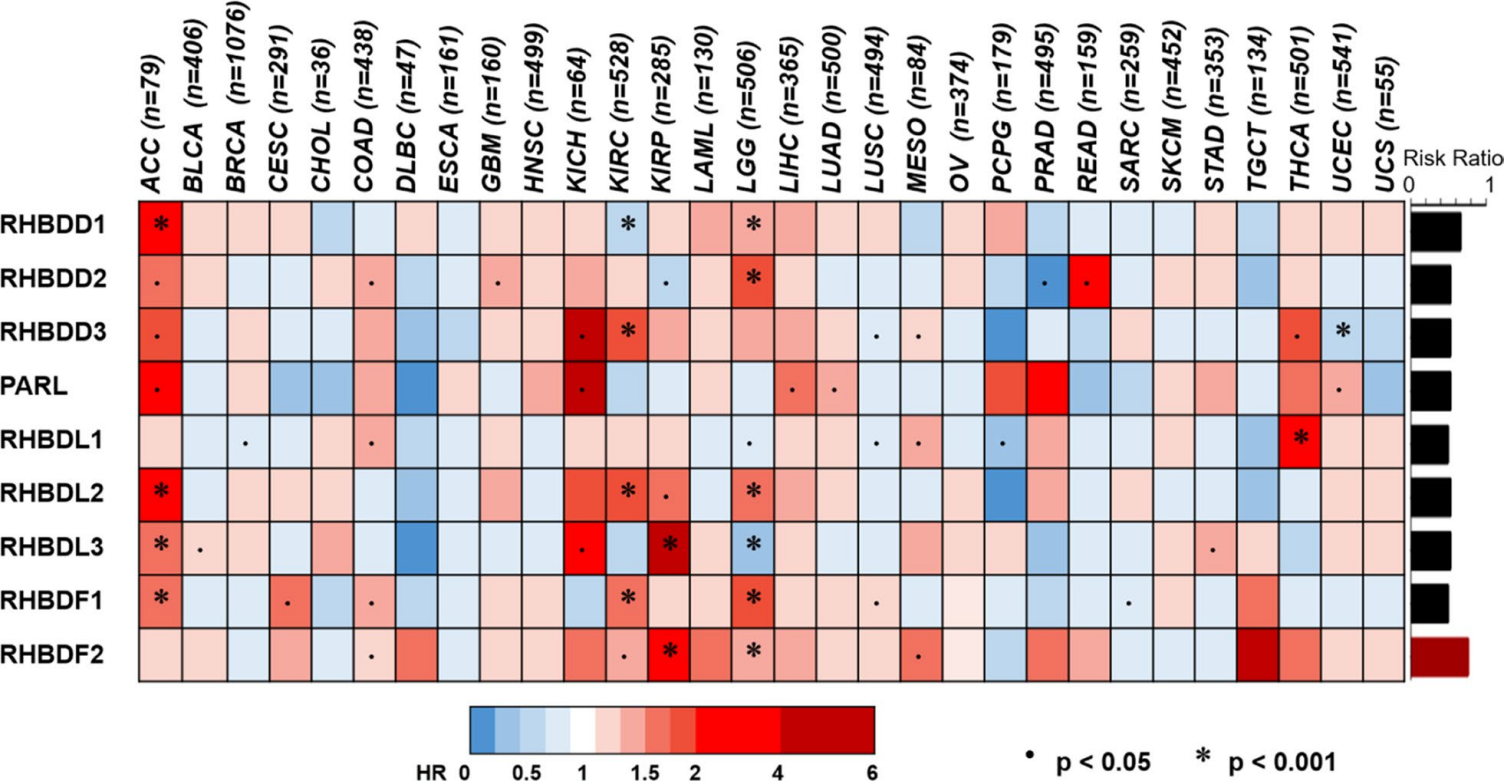
Hazard analysis of rhomboid superfamily in different types of cancers. Heatmap depicted the risk of rhomboid proteins in cancers. (Overall survival data of cancers in TCGA datasets were analyzed, *P*-values were from the log-rank test). Columns at the right side of figure show proportion of poor roles for each gene

**Fig. 2:**
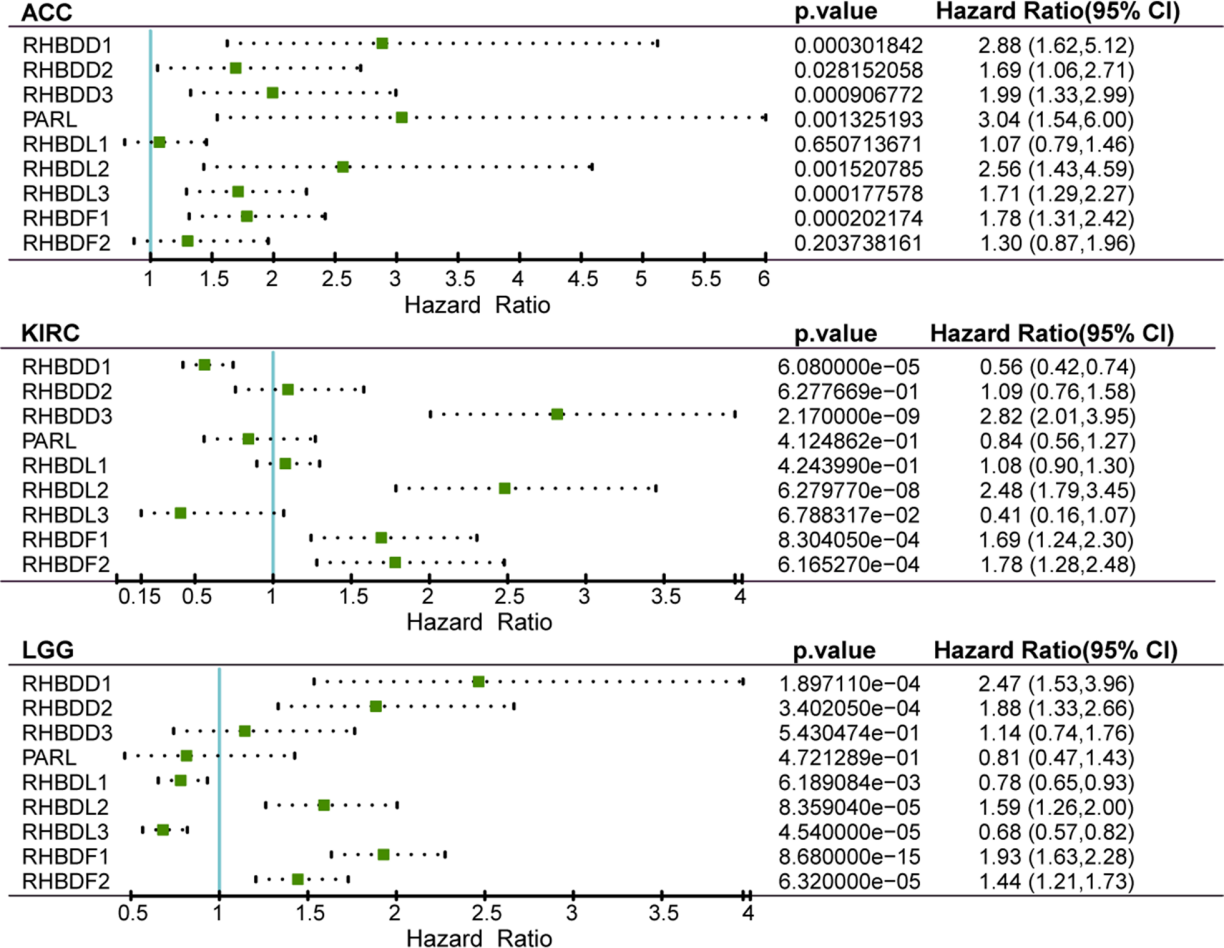
Univariate Cox regression analysis of rhomboid proteins in ACC, KIRC and LGG. Forest plots illustrate the HR, 95% confidence interval and *P*-value of rhomboid proteins in ACC, KIRC and LGG

In order to begin to explore potential correlation between rhomboid functions and cancers, we carried out the univariate Cox regression analysis of the rhomboids family of genes in 30 cancers from TCGA database. The value of Hazard Ratio (HR) was used to describe the influence of the gene expression on tumors (Fig. 1). We found that the expression of rhomboids was most significantly correlated with three types of cancers, namely ACC, LGG and KIRC. The results of HR (Fig. 2); (more details are given in Additional file 1: Table S1) and gene expression patterns analyzed by GEPIA (Additional file 2: Fig.S1) demonstrated that four genes, RHBDD3, RHBDL2, RHBDF1 and RHBDF2, exhibited prognostic value in KIRC. Additionally, high levels of RHBDD1 and RHBDF2 are correlated to poor prognosis of LGG, whereas RHBDD2 and PARL are markedly correlated to ACC disease progression. More importantly, most members of the rhomboid family displayed negative effect on KIRC prognosis. It is worthwhile to point out that RHBDF2 expression is significantly up-regulated in KIRC, in comparison to other rhomboids, suggesting a potential prognostic value.

### Validation the prognostic role of RHBDF2 in KIRC

**Fig. 3:**
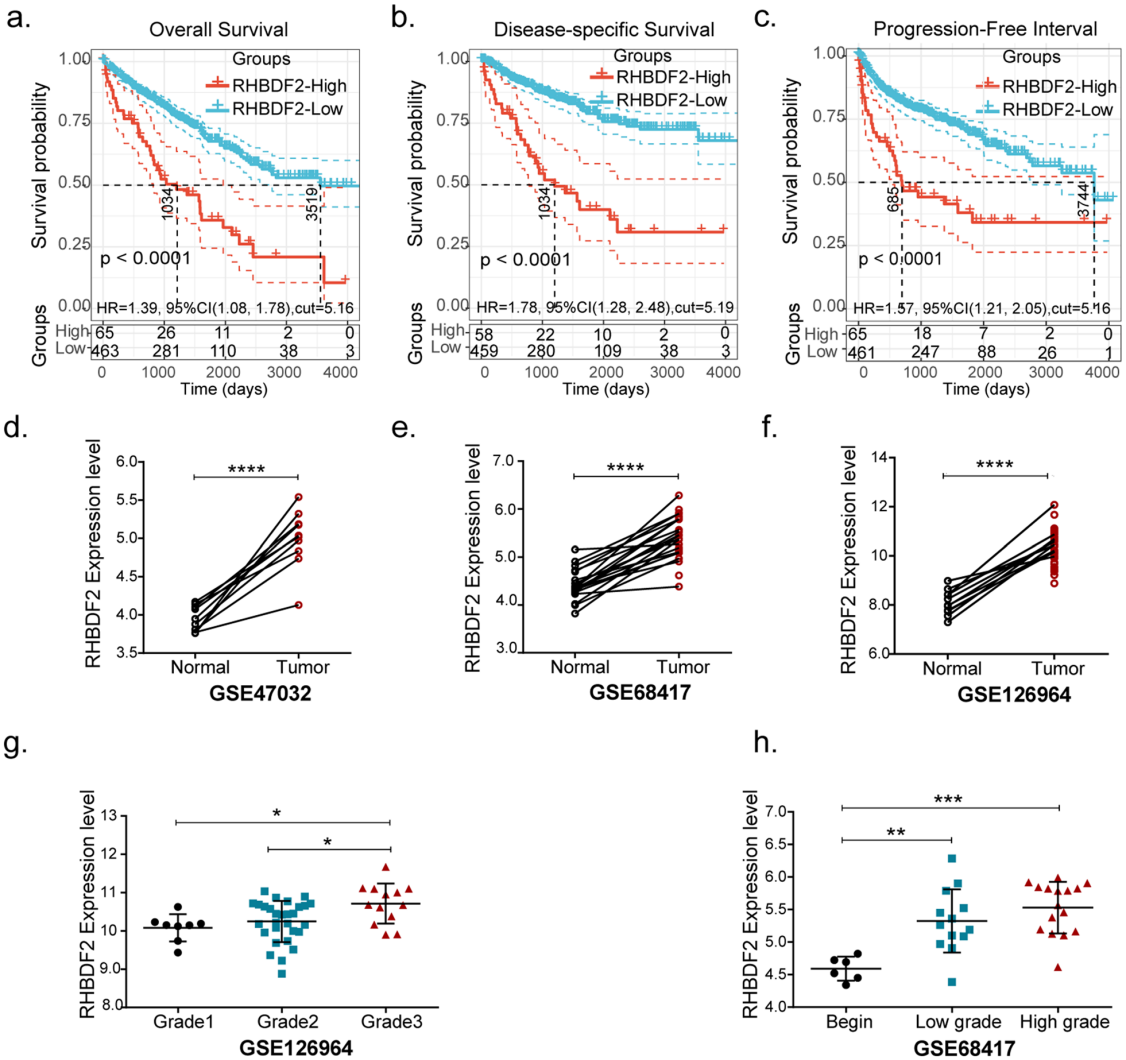
Association between RHBDF2 expression and tumor malignancy in KIRC. Survival analysis of RHBDF2 in KIRC was performed with data in TCGA database. **a** Overall survival analysis. **b** Disease-specific survival analysis. **c** Progression-free interval analysis were present between RHBDF2-high and -low expression groups (*P*-value was from the log-rank test). **d**–**f** RHBDF2 expression between normal and tumor tissues in datasets GSE47032, GSE68417 and GSE126964 respectively (GSE47032, n = 20; GSE126964, n = 66; GSE68417, n = 49; *P*-value was from the paired t-test). **g**, **h** RHBDF2 expression with different tumor grades in GSE126964 and GSE68417 (*P*-value was from one-way ANOVA; * *p* < 0.05, ** *p* < 0.01, *** *p* < 0.005, **** *p* < 0.001)

**Fig. 4:**
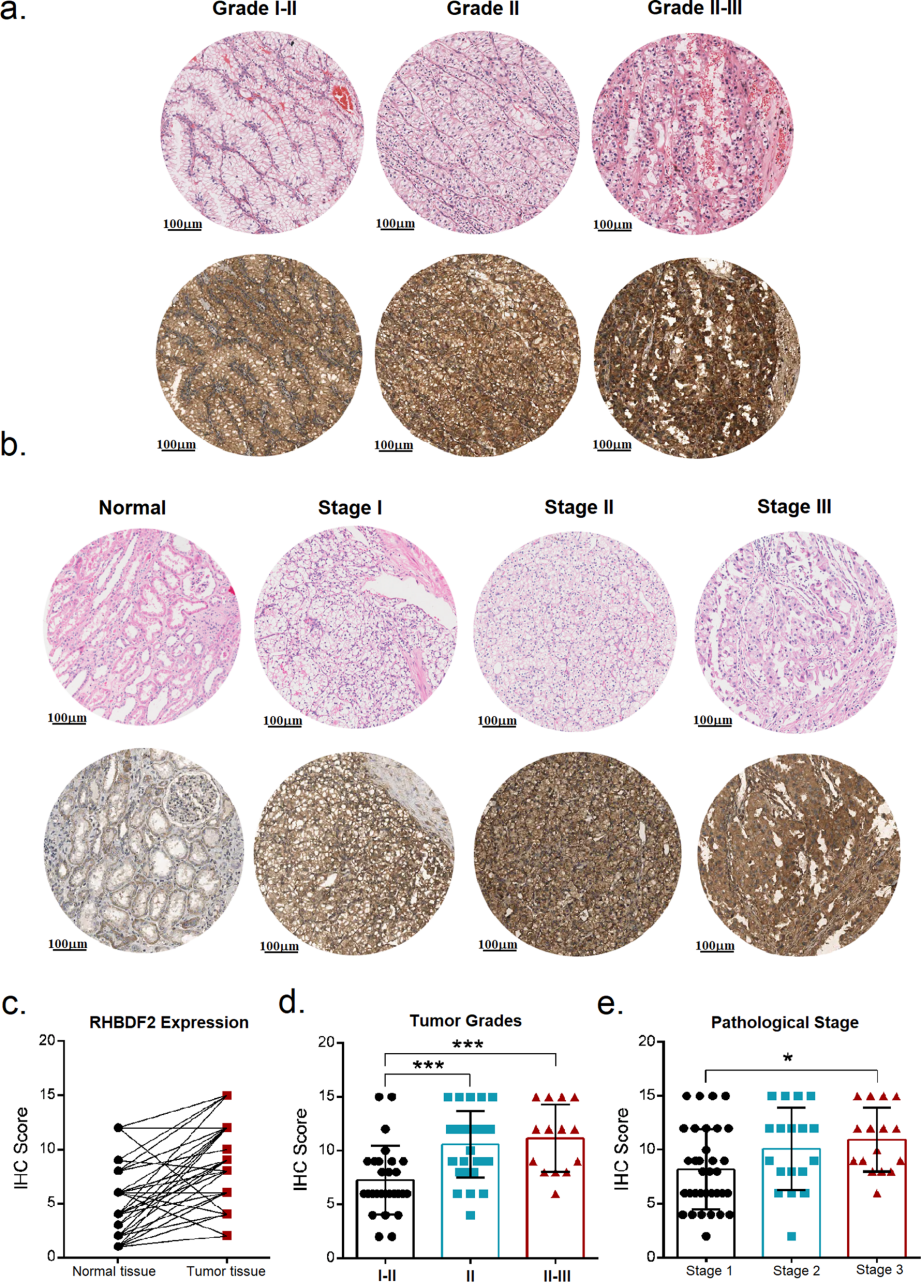
Immunohistochemical staining of RHBDF2 in KIRC tumors with different clinical parameters. **a** H&E and RHBDF2 staining in KIRC tumors with different grades. **b** H&E and RHBDF2 staining in KIRC patients with different pathological stages. **c** Statistics of RHBDF2 staining in tumor and adjacent tissues (n = 75, *P*-value was from the paired t-test). **d**, **e** Statistics of RHBDF2 staining in patients with different tumor grades or pathological stages (n = 70, *P*-value was calculated by one-way ANOVA, * *p* < 0.05, ** *p* < 0.01, *** *p* < 0.005, **** *p* < 0.001)

We then focused on the prognostic value of RHBDF2 for KIRC (Fig. 3a–c). We found that patients in RHBDF2-high group exhibited markedly shorter overall survival (OS), disease-specific survival (DSS), and progression-free survival (PFS) rate. Additionally, we analyzed the relationship between RHBDF2 and clinical parameters recorded in TCGA database. The diagnosed ages and T, N, M stages showed two distinctive differences in RHBDF2-high and low groups. Larger proportion of patients younger than 60-year-old or patients with advanced cancers had higher expression level of RHBDF2 (Table 1). Meanwhile, RHBDF2 had an increased expression in tumor tissue, which was validated in datasets GSE47032, GSE68417, and GSE126964 (Fig. 3d–f) and that high RHBDF2 levels were positively correlated with tumor grades (Fig. 3g, h). We performed immunohistochemical staining in the specimens of KIRC patients with different stages and tumor grades, and found that the RHBDF2 protein was substantially more abundant in high-grade tumors (Fig. 4).

**Table 1:** Clinical characteristics of patients according to RHBDF2 expression in KIRC-TCGA

Characteristics	Total number	RHBDF2 Expression in Groups		*P*-value
		Low (%)	High (%)	
Laterality				
Left	249	117 (44.15%)	132 (50.00%)	ns
Right	280	148 (55.85%)	132 (50.00%)	
Age				
≤ 60	261	113 (42.64%)	148 (55.85%)	< 0.01
> 60	269	152 (57.36%)	117 (44.19%)	
Pathologic T				
T1–2	350	186 (67.64%)	164 (61.89%)	< 0.001
T3–4	190	89 (32.36%)	101 (38.11%)	
Pathologic N				
M0	420	225 (87.89%)	195 (80.58%)	< 0.02
M1	78	31 (12.11%)	47 (19.42%)	
Pathologic stage				
Stage I	265	143 (54.17%)	122 (46.39%)	< 0.05
Stage II–IV	265	121 (45.83%)	141 (53.61%)	

*P*-value was from a Chi-square test

### RHBDF2 overexpression in KIRC correlates with an immunosuppressive microenvironment

**Fig. 5:**
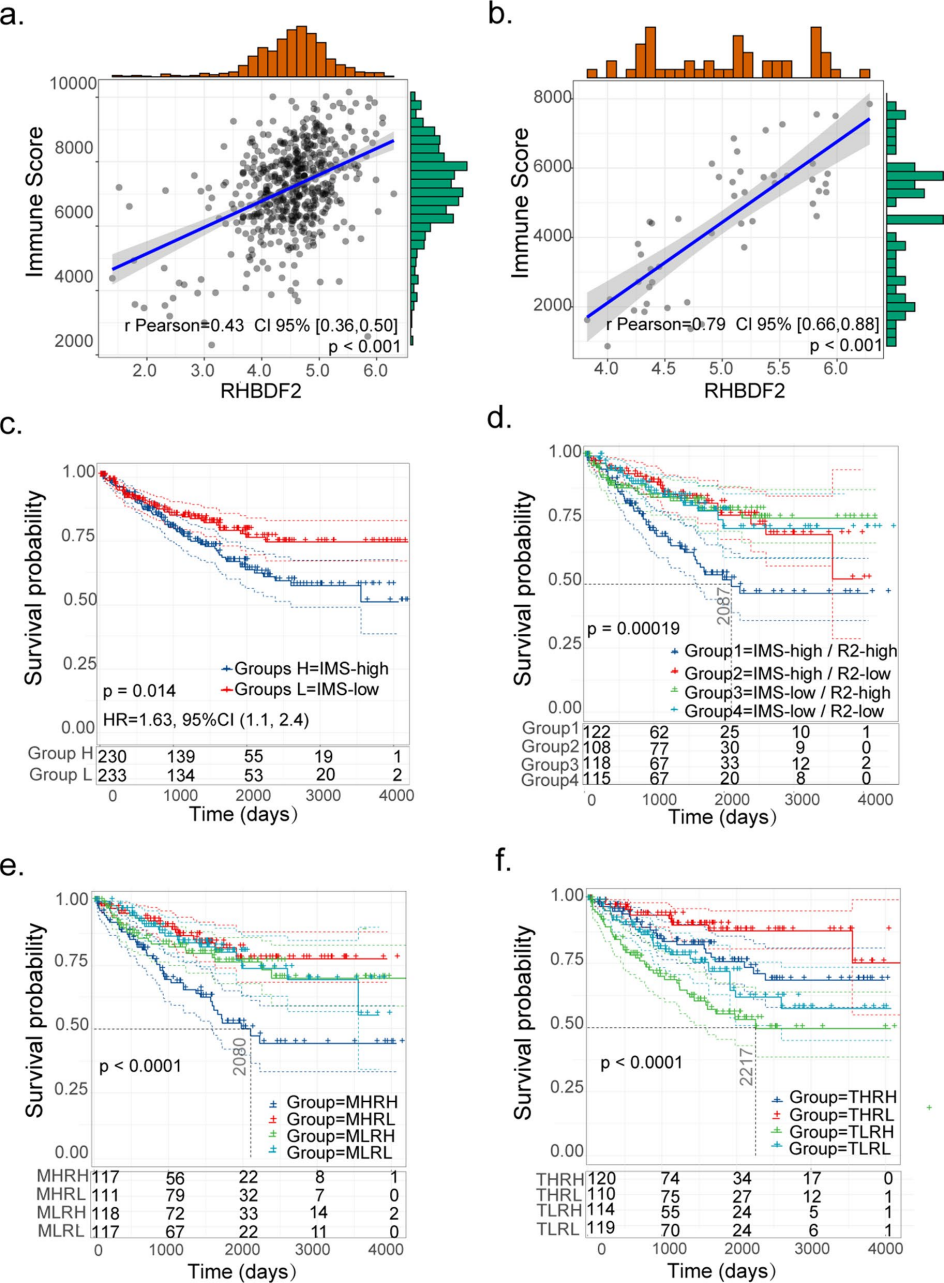
Immune cell infiltration and RHBDF2 expression in survival analysis. Scatter diagram depicted the correlation between immune infiltration scores and RHBDF2 expression with **a** KIRC samples in TCGA database and **b** in GSE68417. **c** Survival analysis for patients with high or low immune infiltration. **d** Survival analysis grouping with immune infiltration scores and RHBDF2 expression. **e** Survival analysis based on the macrophage infiltration scores and RHBDF2 expression (MHRH: macrophage-high infiltration and RHBDF2-high expression; MHRL: macrophage-high infiltration and RHBDF2-low expression; MLRH: macrophage-low infiltration and RHBDF2-high expression; MLRL: macrophage-low infiltration and RHBDF2-low expression). **f** Disease specific survival analysis were present, which grouped with CD8^+^ T cell infiltration and RHBDF2 expression (THRH: CD8^+^ T cell-high infiltration and RHBDF2-high expression; THRL: CD8^+^ T cell-high infiltration and RHBDF2-low expression; TLRH: CD8^+^ T cell-low infiltration and RHBDF2-high expression; TLRL: CD8^+^ T cell-low infiltration and RHBDF2-low expression). *P*-values were from log-rank tests. Data in figure c-f were derived from TCGA

Since KIRC has a character of high lymphocytes infiltration (31), we analyzed the immune infiltration levels in the KIRC samples with gene expression data in TCGA database and GSE68417 using R package “ESTIMATE”. And the results of correlation analysis showed that RHBDF2 was positively correlated with enhanced immune infiltration (Fig. 5a, b). We then divided the KIRC samples into immune infiltration score-high (IMS-high) and -low (IMS-low) groups, and carried out survival analysis. We found that KIRC patients in the IMS-high group exhibited a shorter survival period compared to those in the IMS-low group (Fig. 5c). We further divided the KIRC samples into four groups: immune infiltration-high and RHBDF2 expression-high (IMS-high/R2-high), IMS-high/R2-low, IMS-low/R2-high and IMS-low/R2-low. Disease specific survival analysis of these specimens indicated that patients in IMS-high/R2-high group had the shortest survival time (Fig. 5d).

It was reported previously that KIRC patients with high macrophage and CD8^+^ T cell infiltration survived poorly [53]. We scored the infiltrated lymphocytes by TIMER 2.0 with data in KIRC-TCGA and conducted survival analyses based on immune infiltration scores and RHBDF2 expression levels. We found that patients with both high level of macrophages infiltration and RHBDF2 expression suffered shorter survival time than those in other groups (Fig. 5e). Additionally, patients with higher CD8^+^ T cell infiltration and lower RHBDF2 expression exhibited favorable prognosis, whereas patients in higher CD8^+^ T cell infiltration and higher RHBDF2 expression group exhibited the poorest prognosis (Fig. 5f). These findings suggest that high levels of RHBDF2 prominently affect the anti-cancer activities of macrophages and CD8^+^ T cells.

### RHBDF2 gene-silencing leads to reduction of PD-L1 level in renal cancer cells

Overexpressed RHBDF2 inhibited the function of infiltrated immune cells, which suggests us to analyze the expression of immune checkpoints molecules. We evaluated immunosuppressive checkpoints expression in tumor tissues and normal tissue. *CD274, CD273, VTCN1, CD276, LGALS9* and *CMTM4* were up-regulated in KIRC tissues (Additional file 3: Fig. S2). Then we analyzed the Pearson correlation between those checkpoints and *RHBDF2* in TCGA and GEO datasets. The expression of *CD273, CD276* and *LGALS9* had stronger positive correlation with *RHBDF2*. The correlation of *RHBDF2* between *CD274* and *CMTM6* were also light. However, *VTCN1* and *CMTM4* were negatively correlated to *RHBDF2* (Additional file 4: Fig. S3a). Positive correlation of *CD273* and *CD276* with the expression of *RHBDF2* was detected in 786-O and 769-P cells by RT-qPCR (Additional file 4: Fig. S3b).

**Fig. 6:**
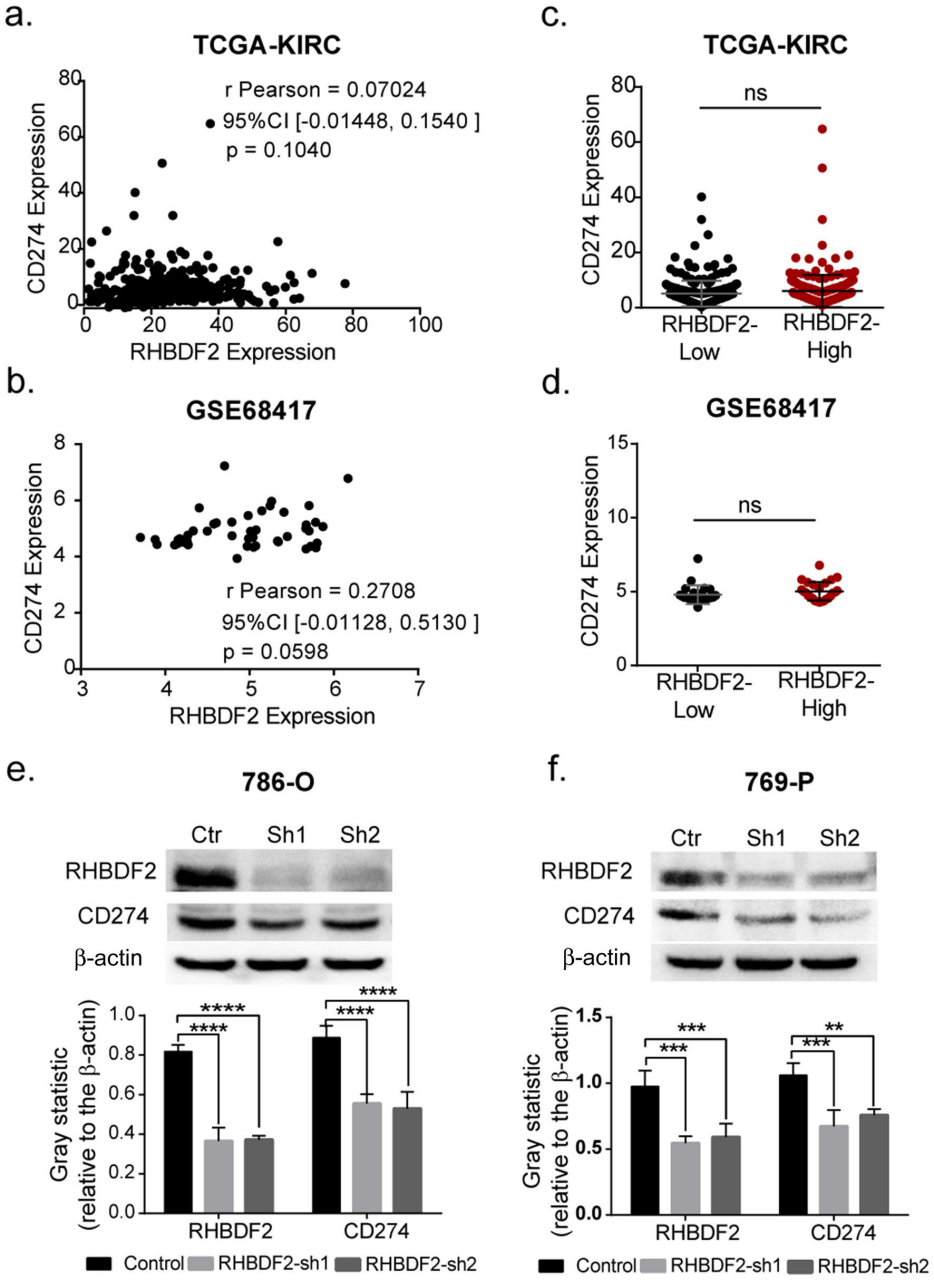
Immune checkpoints analysis. **a** Pearson correlation between *CD274* and *RHBDF2* expression in renal clear cell carcinoma in TCGA database and in **b** GSE68417 datasets. **c** RNA sequencing dada of *CD274* expressing in renal clear cell carcinoma in TCGA database (RHBDF2-low, n = 269; RHBDF2-high, n = 269; unpaired t-test) and in **d** GSE68417 datasets (RHBDF2-low, n = 24; RHBDF2-high, n = 25; unpaired t-test). **e**, **f** Protein levels of PD-L1 in RHBDF2-knockdown cell lines, 786-O and 769-P (*P*-values were from one-way ANOVA, * *p* < 0.05, ** *p* < 0.01, *** *p* < 0.005, **** *p* < 0.001)

PD-L1, the product of the *CD274* gene, is an immune checkpoint protein highly expressed in renal carcinoma [54]. We measured the transcription relevance between *RHBDF2* and *CD274* in the databases but found no significant correlations (Fig. 6a–d). We then prepared shRNA lentivirus to artificially knockdown RHBDF2 in renal cancer cell-lines (786-O and 769-P). We found that protein level of PD-L1 decreased in RHBDF2 knocked-down cells (Fig. 6e–h). These findings suggested that RHBDF2 may have a potentially important role in the maintenance of immune checkpoints level in renal cancer cells, which could make RHBDF2 a valuable target in assisting immunotherapy.

### Key modules identification and functional annotation analysis

**Fig. 7:**
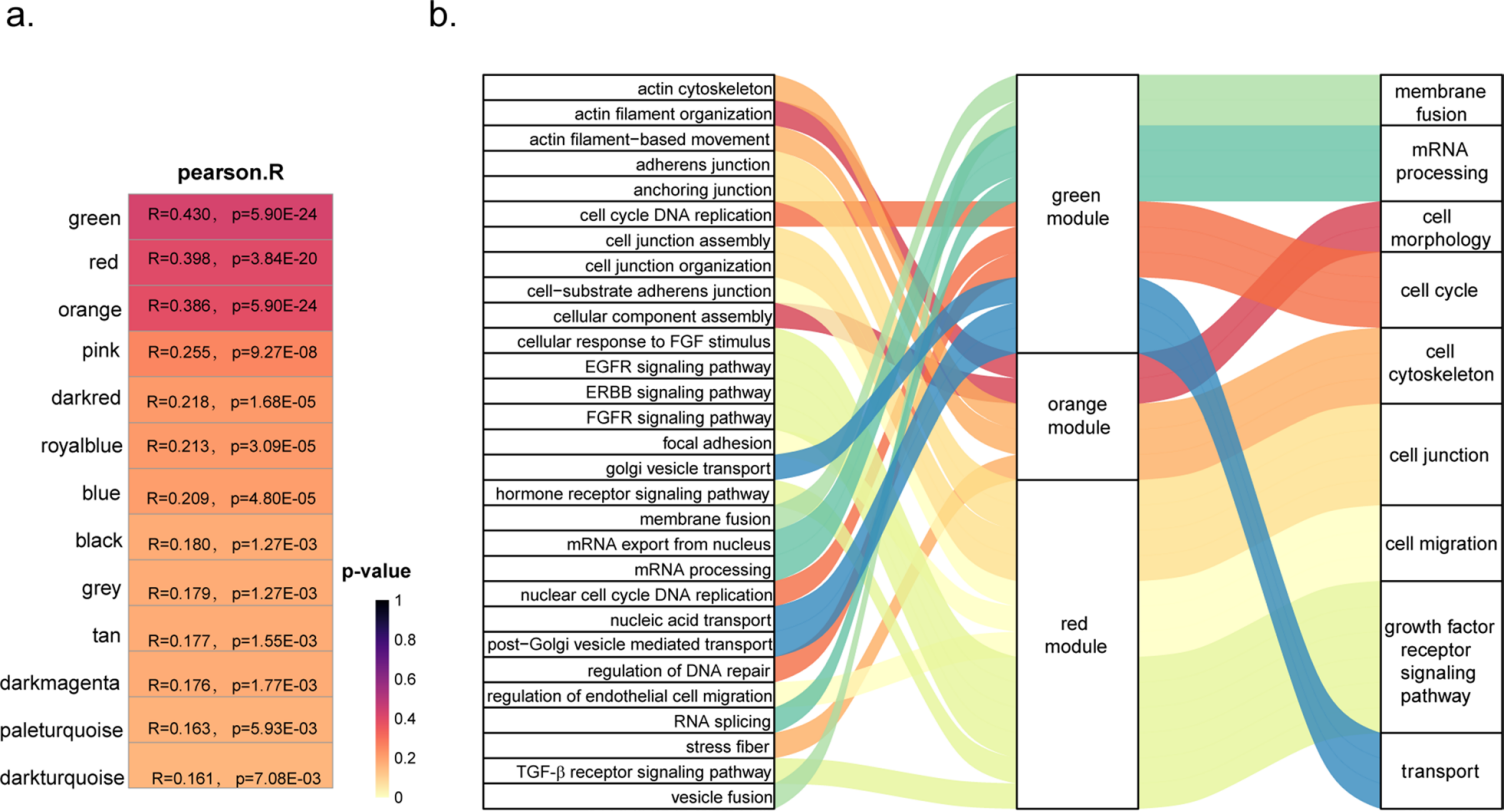
RHBDF2-related function enrichment. **a** Relationships between modules and trait analyzed by WGCNA. RHBDF2 expression level was set as a trait, genes in green, red and orange modules had stronger correlation with *RHBDF2* expression. **b** Functional annotation of genes in green, red and orange modules

In order to further investigate the biological characteristics of renal clear cell carcinoma that are associated with various levels of RHBDF2 expression, we carried out WGCNA. Messenger RNA profiles of the specimens with similar patterns were grouped into several functional modules based on WGCNA calculation. These operations revealed a total of 53 modules (Additional file 5: Fig. S4a). The transcription level of RHBDF2 was used as a characteristic trait for grouping. We determined the relevance between each functional module and grouping trait by Pearson correlation analysis, and identified the top three modules correlated with RHBDF2 level and marked them, respectively, in green, red and orange (Fig. 7a, Additional file 5: Fig. S4b-c, Additional file 6: Table S2). We determined the hub genes in the green, red and orange modules by using Network Analyst (Additional file 7: Table S3). The hub genes in each module were used to carry out the gene-interactive network analysis and annotate the module-related functions by GeneMANIA. The results of gene network analyses were shown in Additional file 8: Fig. S5, Additional file 9: Fig. S6, Additional file 10: Fig. S7. Functional annotation of hub module genes was shown in a bubble diagram (Additional file 11: Fig. S8). The function of orange module genes was related to the cell morphology and cell cytoskeleton, the function of green module genes was related to mRNA processing, membrane fusion and cell cycle, and the genes in red module mostly show connections with cell junction, cell migration and growth factor receptor signaling pathways (Fig. 7b).

### *RHBDF2* gene-silencing restricts renal clear cell cancer cell proliferation and migration

**Fig. 8:**
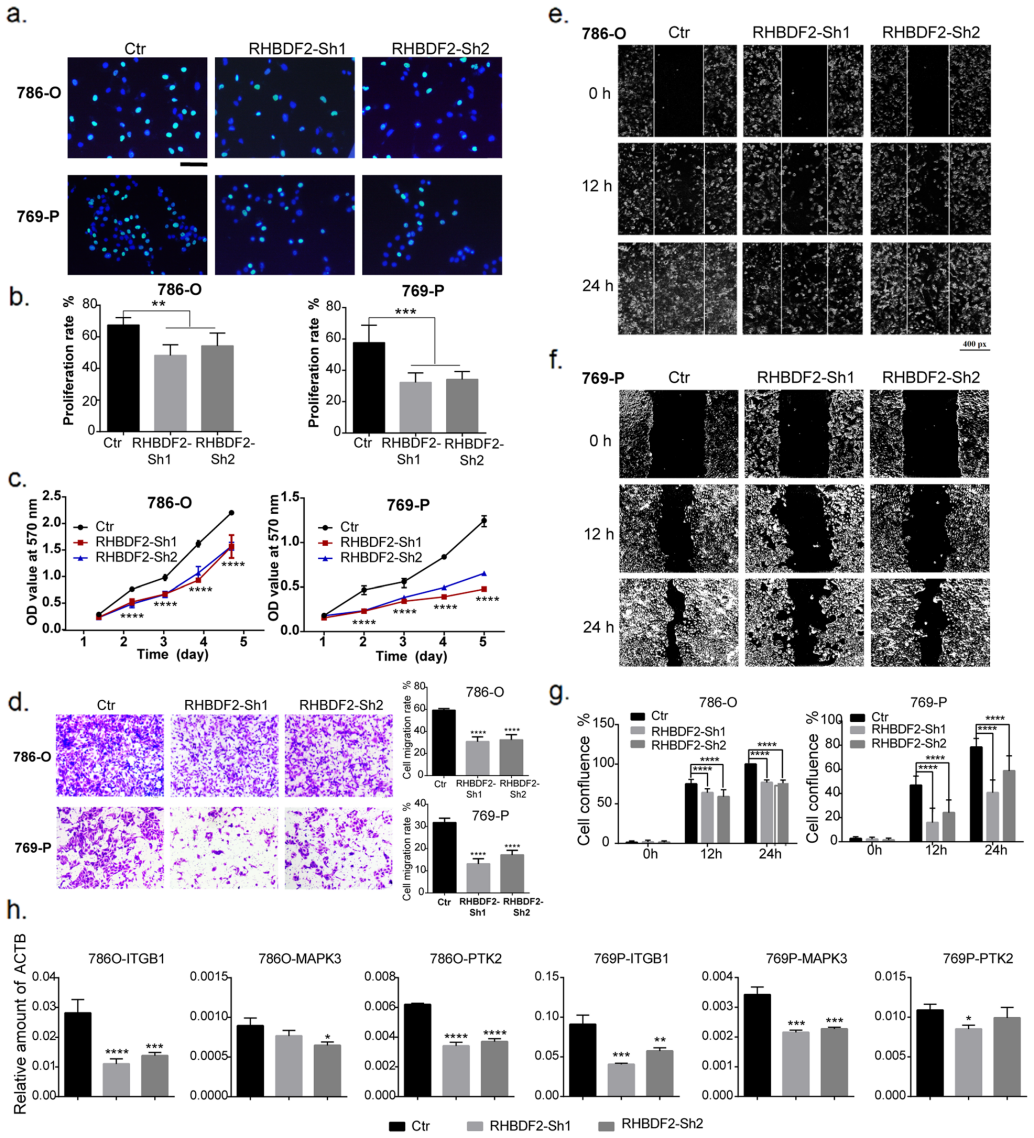
RHBDF2 knockdown restricted renal clear cell tumor proliferation and migration. **a** Cell proliferation detection by Edu incorporation assay in 786-O cells and 769-P cells with or without *RHBDF2* knockdown. (Blue: nuclei; green: nuclei of S-phase cells.) **b** Statistics of EDU incorporation rate (t-test). **c** Statistics of Cell growth speed (one-way ANOVA was used for significance test). **d** Cells with or without RHBDF2 knockout were used to evaluate migration ability by Transwell assay. The statistical results were shown on the right side (one-way ANONA). **e** Cell healing results of 786-O cells and **f** 769-P cells, with or without RHBDF2 knockdown at 0, 12, 24 h after scratching. **g** Statistics of scratching healing (two-way ANOVA). **h** mRNA expression of hub genes in 786-O cells and 769-P cells with or without RHBDF2 knockdown (one-way ANOVA). All the results were repeated three times, * *p* < 0.05, ** *p* < 0.01, *** *p* < 0.005, **** *p* < 0.001

To verify the results from bioinformatics analysis that the genes and pathways related to RHBDF2 functions may contribute to cancer cell migration and signal transduction, we silenced the *RHBDF2* gene by using shRNA in renal clear cell carcinoma cell line 786-O and 769-P. The cell-cycle was measured by Edu-incorporation in which all cell nuclei were stained blue with Hoechst33342 and the nuclei of cells in S-phase were labeled with green fluorescence. We found that the proportion of green fluorescence decreased in *RHBDF2* gene knocked-down cells, indicating a decreased proliferation rate (Fig. 8a, b). The decrease of cell proliferation rate as a result of RHBDF2 knockdown was verified by MTT assay (Fig. 8c). Moreover, we carried out trans-well and scratch healing assays, and found that RHBDF2 knockdown gave rise to significantly reduced cell motility in 786-O cells and 769-P cells in comparison with the scramble shRNA treated cells (Fig. 8d–g). Furthermore, we used quantitative real-time PCR to detect migration related hub genes obtained by enrichment analysis, and found that the transcription of ITGB1, MAPK3 and PTK2 were significantly decreased in RHBDF2 knocked-down cells (Fig. 8h).

**Fig. 9:**
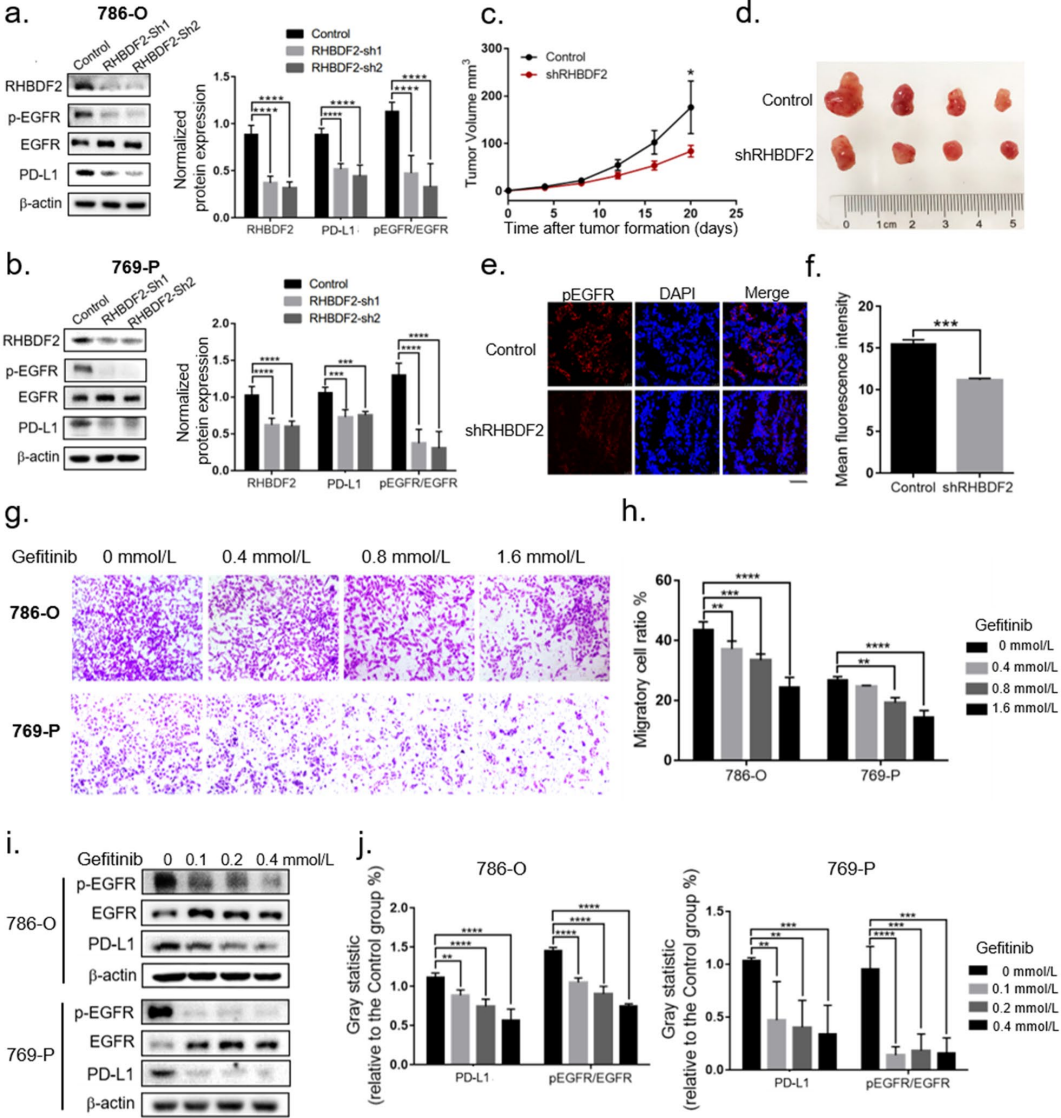
RHBDF2 related functions were mediated by EGFR signaling pathway. **a**, **b** Phosphorylation of EGFR and PD-L1 protein level in 786-O and 769-P cells were detected by western blot. Significance testing of gray statistics was analyzed by two-way ANOVA. **c** Growth rate of the transplanted tumor in the control group and RHBDF2 knockdown group (data were presented as the mean ± SEM and subjected to two-way ANOVA for significance test). **d** Representative images of xenografts in nude mice. **e** Immunofluorescent staining of phosphorylation of EGFR in the graft sections (The horizontal line at the bottom right represents 50 microns). **f** The mean fluorescence intensity of the phosphorylation staining of EGFR in the graft sections (t-test). **g** The migratory ability testing of 786-O cells and 769-P cells after Gefitinib treatment. **h** Statistics of the cell migratory ability after Gefitinib treatment (two-way ANOVA). **i** The detection of pEGFR and PD-L1 level in 786-O and 769-P cells after Gefitinib treatment. **j** Gray statistics of pEGFR and PD-L1 level in 786-O and 769-P cells (two-way ANOVA, * *p* < 0.05, ** *p* < 0.01, *** *p* < 0.005, **** *p* < 0.001)

To investigate the function of RHBDF2 in vivo, we constructed a xenograft model based on the KIRC cell line 786-O in nude mice. As shown in Fig. 9c, d, the tumor growth rate in the RHBDF2-knockdown group was significantly slower than that in the control group. At the same time, we also observed a delay in the early stage of tumor formation in RHBDF2-knockdown group.

### RHBDF2 related functions were mediated by EGFR signaling pathway

We then focus on the signaling that RHBDF2 mediated. We used the data of KIRC-TCGA to carry out the GSEA, combined the results with WGCNA and obtained the functional enrichment results of RHBDF2 in both analysis (Additional file 12: Table S4). We found the enrichment results of the red module in WGCNA were more similar to those of GSEA (Additional file 13: Fig. S9). Also, as shown in Fig. 7b, we presented the pathways that RHBDF2 possibly regulated, the enriched ones of which contained EGFR, ERBB and FGFR. In Fig. S5, the hub genes of the red module, like PTK2, MAPK3, were also connected to the EGFR pathway, as a role of the downstream of signaling or interaction, which suggested the correlation of RHBDF2 and EGFR. Meanwhile, we detected the reduction of phospho-EGFR in RHBDF2 knockdown cells (Fig. 9a, b), and the phenomenon was also remarkable in the tumor sections of 786-O xenografts (Fig. 9e, f). We used EGFR inhibitor Gefitinib to significantly reduce the migration ability of 786-O and 769-P cells (Fig. 9g, h), which was consistent with the effect of RHBDF2-knockdown treatment. Based on the above results, we speculate that the EGFR signaling pathway may be the main mediator of RHBDF2's regulation on cell invasion and migration.

EGFR pathway was also reported to regulate PD-L1 level [55]. When we used Gefitinib to block EGFR signaling, the protein level of PD-L1 showed a reduction (Fig. 9i, j). In view of RHBDF2 knockdown significantly reduced EGFR activation, it’s plausible that EGFR pathway was an important signaling pathway by which RHBDF2 regulates PD-L1.

## Discussion

The primary goal of this study is to gain initial insights into the role of the rhomboid family of genes in cancer progression in general, and that of the inactive rhomboids more specifically. The challenge from this study is the need to integrate a large amount of prognostic information of 30 cancers and the expression abundance of rhomboids in different cancers. The approach leads us to a more holistic understanding of the role of rhomboids in cancer. Our findings indicate that KIRC disease progression is highly likely to be affected by fluctuations of rhomboids gene expressions and, more specifically, by high levels of RHBDF2 gene expression. And RHBDF2 is a good indicator of poor prognosis of the disease. Advanced analyses of KIRC, like WGCNA, GO/KEGG pathways annotation pointed to the regulatory role of RHBDF2 in cell proliferation and migration, implicating its potential as a target for cancer therapy.

The result of immune infiltration analysis is interesting, high levels of RHBDF2 positively correlates with infiltration of lymphocytes in cancer tissues but is not conductive to the survival of patients. By analyzing the immune suppressive checkpoints level, we confirmed that RHBDF2 show positive correlation with checkpoints, like CD273 and CD276. Moreover, we found that RHBDF2 functions may be critically required in establishing high PD-L1 protein levels in cancer cells, which suggests that RHBDF2 be a valuable therapeutic target in line with PD-L1-focused immunotherapy, in addition to being of diagnostic and prognostic factor for renal clear cell cancer. The question of enhanced immune infiltration into the tumors is particularly interesting. A previous report of KIRC tumors [56] identified three distinct groups, namely immune silencing, immune activation and immune regulation, based on the infiltration of CD8^+^ T cells. T cells in the immune-regulated group are less clonal and less cytotoxic than those in the immune-activated group. It is plausible that RHBDF2 over-expression encourages the formation of an immunosuppressive environment. In a recent study [57], we also found that RHBDF1, another member of the proteolytically inactive rhomboid, affected tumor immune microenvironment, through a promotion of endothelial-mesenchymal transition and tumor fibrotic stroma growth. Targeting RHBDF2 either at gene expression or protein function could lead to a release of immunosuppression in tumors and thereby a possible enhancement of the cytotoxicity of immune cells such as macrophages and T-cells.

In future studies, mechanisms underlying the role of RHBDF2 in the modulation of renal clear cell tumor progression and potential impact on the microenvironment are of particular interest. Additionally, in-depth understanding of the plausible linkage between RHBDF2 and PD-L1 is worthy exploration. Such studies may include evaluating the responses to PD-L1 treatment in patients with various levels of RHBDF2. Moreover, RHBDF2 as a relatively large protein molecule and the possibility that it may interact with a number of proteins with critical functions make it an excellent target for therapy development.

## Conclusions

In summary, we found that RHBDF2 is positively correlated with the severity of the malignancy of renal clear cell carcinoma. High expression of RHBDF2 in KIRC is associated with an activation of a number of genes involved in tumor growth and metastasis. Silencing the *RHBDF2* gene in renal cancer cells leads to down-regulation of the immunosuppressive checkpoint protein PD-L1, even though there is an increase of lymphocyte infiltration into the tumors with high levels of RHBDF2. These findings are consistent with the view that RHBDF2 not only has potential diagnostic and prognostic values as biomarker, it may also be of important value as a therapeutic target in assisting immunotherapy.

## Supplementary Information

**Additional file 1: Table S1.** Univariate Cox regression analysis of rhomboid family genes in cancers from TCGA database.

**Additional file 2: Fig. S1.** Expression pattern of rhomboid genes in ACC, KIRC, LGG and their adjacent normal tissues. Expression of rhomboid genes in tumor and corresponding normal tissues in ACC, KIRC and LGG were analyzed by GEPIA with data in TCGA and GTEx databases (ANOVA, * *p* < 0.05).

**Additional file 3: Fig. S2.** Expression of immune checkpoint molecules between tumor tissues and normal tissues in GEO and KIRC-TCGA datasets. The array of GSE167093 was processed using vst transformation and quantile normalization. The array of GSE68417 was processed with quantile normalization and log2-transformation. The data of GSE126964 was normalized to FPKM value and processed with log2-transformation. The data of KIEC-TCGA was normalized to TPM value and processed with log2-transformation. *P*-values were form one-way ANOVA, * *p* < 0.05, ** *p* < 0.01, *** *p* < 0.005, **** *p* < 0.001.

**Additional file 4: Fig. S3.** Immune checkpoints analysis. (a) Pearson correlation of the expression of immune checkpoints and RHBDF2. (* *p* < 0.001, • *p* < 0.01). (b) Detection of the CD273, CD276 and LGALS9 expression in 786-O and 769-P with or without RHBDF2 knockdown (one-way ANOVA, * *p* < 0.05, ** *p* < 0.01, *** *p* < 0.005, **** *p* < 0.001).

**Additional file 5: Fig. S4.** Weighted Gene Correlation Network Analysis. (a) Co-expression modules for the KIRC transcriptome. (b) Module genes connectivity and genes significance in green, red and orange modules were present respectively. (c) Module genes significance for RHBDF2 expression and their module membership in green, red and orange modules were present.

**Additional file 6: Table S2.** Correlation between RHBDF2 level and different modules in WGCNA.

**Additional file 7: Table S3.** Hub genes of red, green and orange modules in WGCNA.

**Additional file 8: Fig. S5.** Gene interactive network of hub genes in red module.

**Additional file 9: Fig. S6.** Gene interactive network of hub genes in green module.

**Additional file 10: Fig. S7.** Gene interactive network of hub genes in orange module.

**Additional file 11: Fig. S8.** Functional enrichment analyses of hub genes in red, green and orange modules.

**Additional file 12: Table S4.** Significant enrichment pathways and functions both in WGCNA and GSEA.

**Additional file 13: Fig. S9.** Numbers of significant enrichment pathways and functions both in WGCNA and GSEA.

## Data Availability

Data in this article were downloaded from publicly available datasets. The datasets for this study can be found in the TCGA database (https://cancergenome.nih.gov/) and GEO database (https://www.ncbi.nlm.nih.gov/gds).

## Abbreviations

ACC: Adrenocortical carcinoma
BLCA: Bladder urothelial carcinoma
BRCA: Breast invasive carcinoma
CESC: Cervical squamous cell carcinoma and endocervical adenocarcinoma
CHOL: Cholangiocarcinoma
COAD: Colon adenocarcinoma
DLBC: Diffuse large b-cell lymphoma
DSS: Disease-specific survival
EGFR: Epidermal growth factor receptor
ESCA: Esophageal carcinoma
GBM: Glioblastoma multiforme
GEO: Gene Expression Omnibus
GEPIA: Gene Expression Profiling Interactive Analysis
GSEA: Gene Set Enrichment Analysis
GTEx: Genotype-Tissue Expression Project
HNSC: Head and neck squamous cell carcinoma
HR: Hazard Ratio
ICI: Immune checkpoint inhibitors
IMS: Immune infiltration score
KICH: Kidney chromophobe
KIRC: Kidney renal clear cell carcinoma
KIRP: Kidney renal papillary cell carcinoma
LAML: Acute myeloid leukemia
LGG: Brain lower grade glioma
LIHC: Liver hepatocellular carcinoma
LUAD: Lung adenocarcinoma
LUSC: Lung squamous cell carcinoma
MESO: Mesothelioma
OS: Overall survival
OV: Ovarian serous cystadenocarcinoma
PCPG: Pheochromocytoma and paraganglioma
PFS: Progression-free survival
PRAD: Prostate adenocarcinoma
READ: Rectum adenocarcinoma
SARC: Rectum adenocarcinoma sarcoma
SKCM: Skin cutaneous melanoma
STAD: Stomach adenocarcinoma
TCGA: The Cancer Genome Atlas
TGCT: Testicular germ cell tumor
THCA: Thyroid carcinoma
UCEC: Uterine corpus endometrial carcinoma
UCS: Uterine carcinosarcoma
WGCNA: Weighted Gene Correlation Network Analysis
