# Supplementary figures and images for: RHBDF2 gene functions are correlated to facilitated renal clear cell carcinoma progression
Source: Cancer Cell Int. 2021 Nov 4;21:590. doi: 10.1186/s12935-021-02277-0 (PMC8567583; doi:10.1186/s12935-021-02277-0)

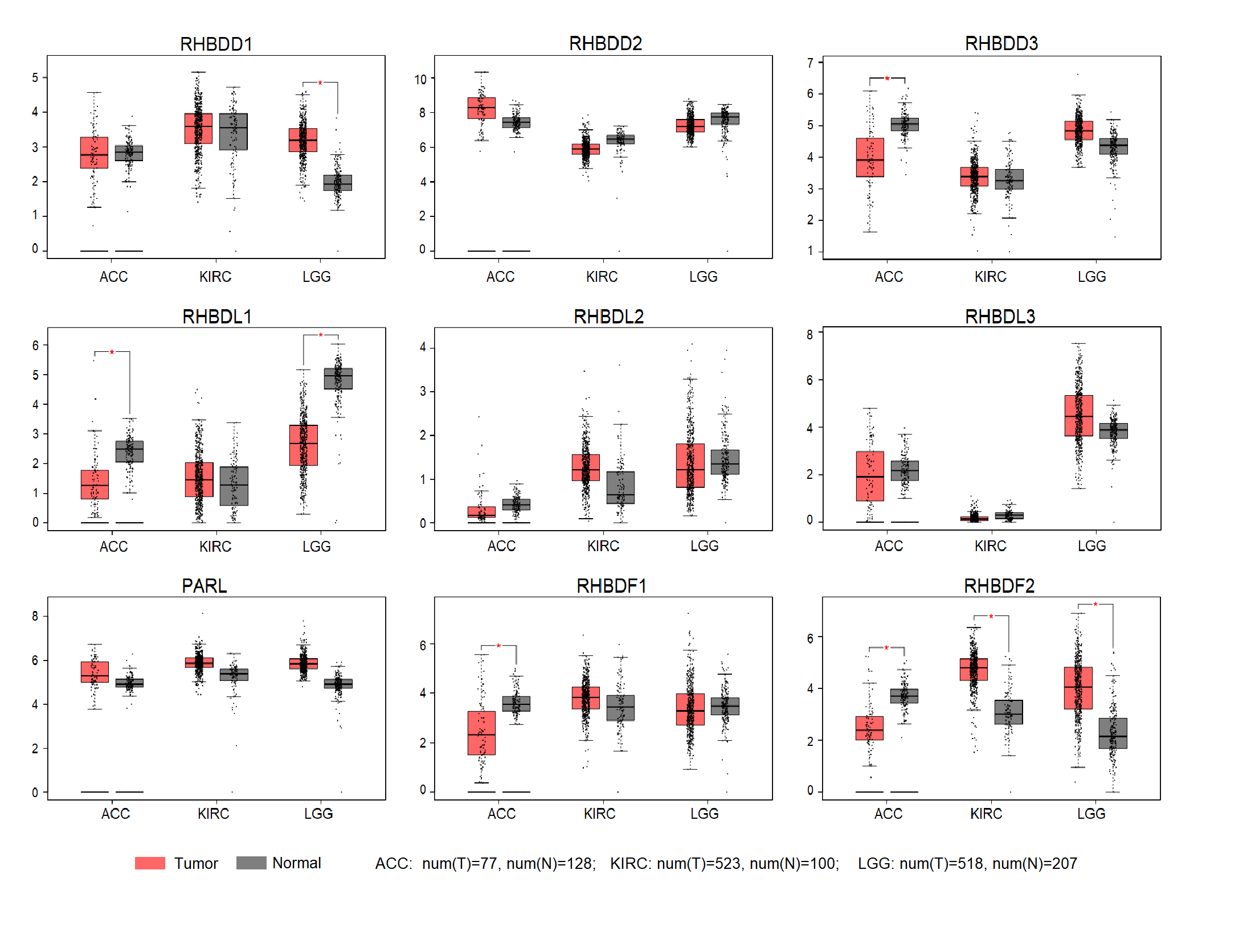

Supplement: Supplementary file 2 — Additional file 2: Fig. S1. Expression pattern of rhomboid genes in ACC, KIRC, LGG and their adjacent normal tissues. Expression of rhomboid genes in tumor and corresponding normal tissues in ACC, KIRC and LGG were analyzed by GEPIA with data in TCGA and GTEx databases (ANOVA, * p < 0.05). [file 12935_2021_2277_MOESM2_ESM.tif]

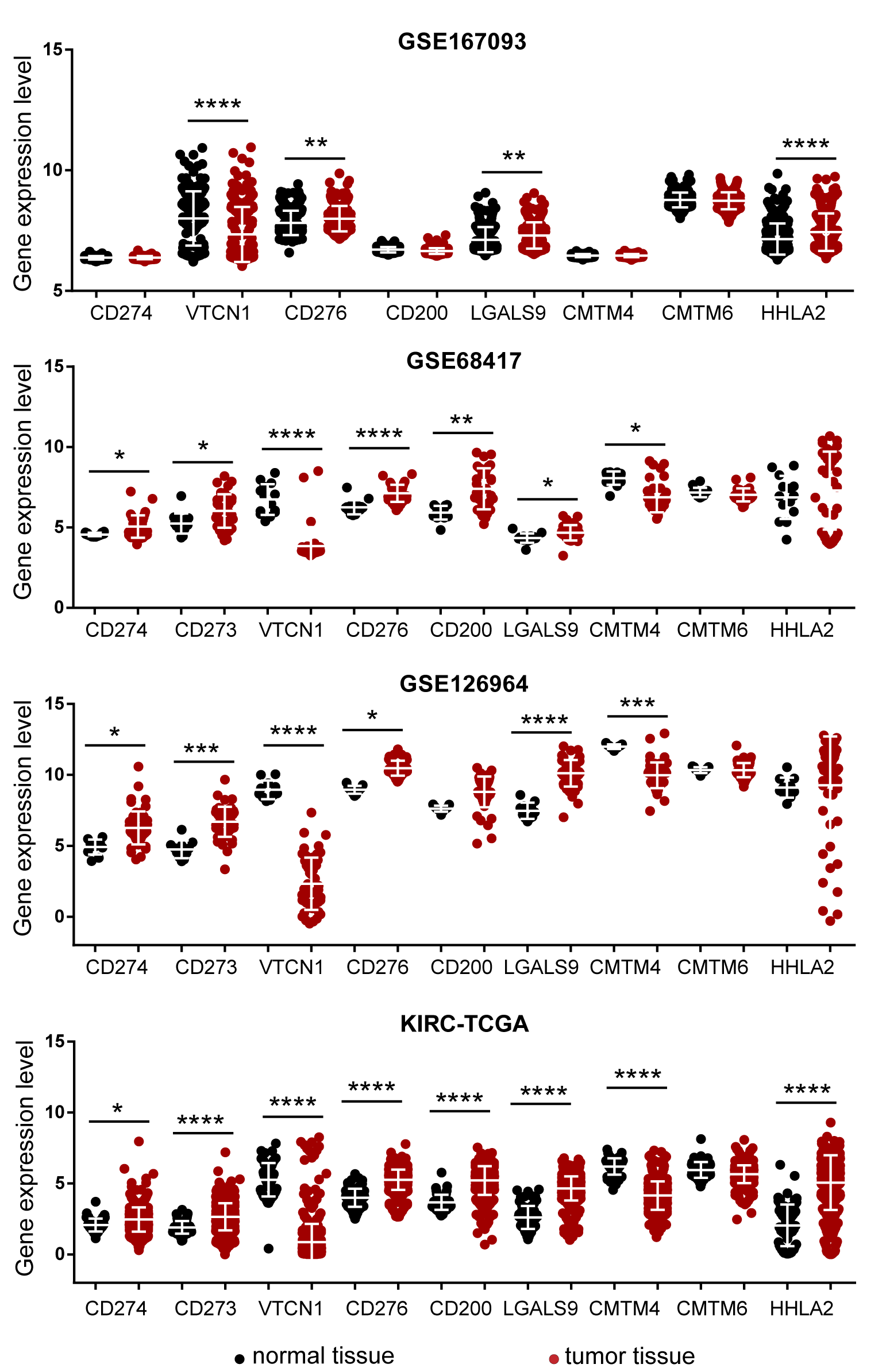

Supplement: Supplementary file 3 — Additional file 3: Fig. S2. Expression of immune checkpoint molecules between tumor tissues and normal tissues in GEO and KIRC-TCGA datasets. The array of GSE167093 was processed using vst transformation and quantile normalization. The array of GSE68417 was processed with quantile normalization and log2-transformation. The data of GSE126964 was normalized to FPKM value and processed with log2-transformation. The data of KIEC-TCGA was normalized to TPM value and processed with log2-transformation. P-values were form one-way ANOVA, * p < 0.05, ** p < 0.01, *** p < 0.005, **** p < 0.001. [file 12935_2021_2277_MOESM3_ESM.tif]

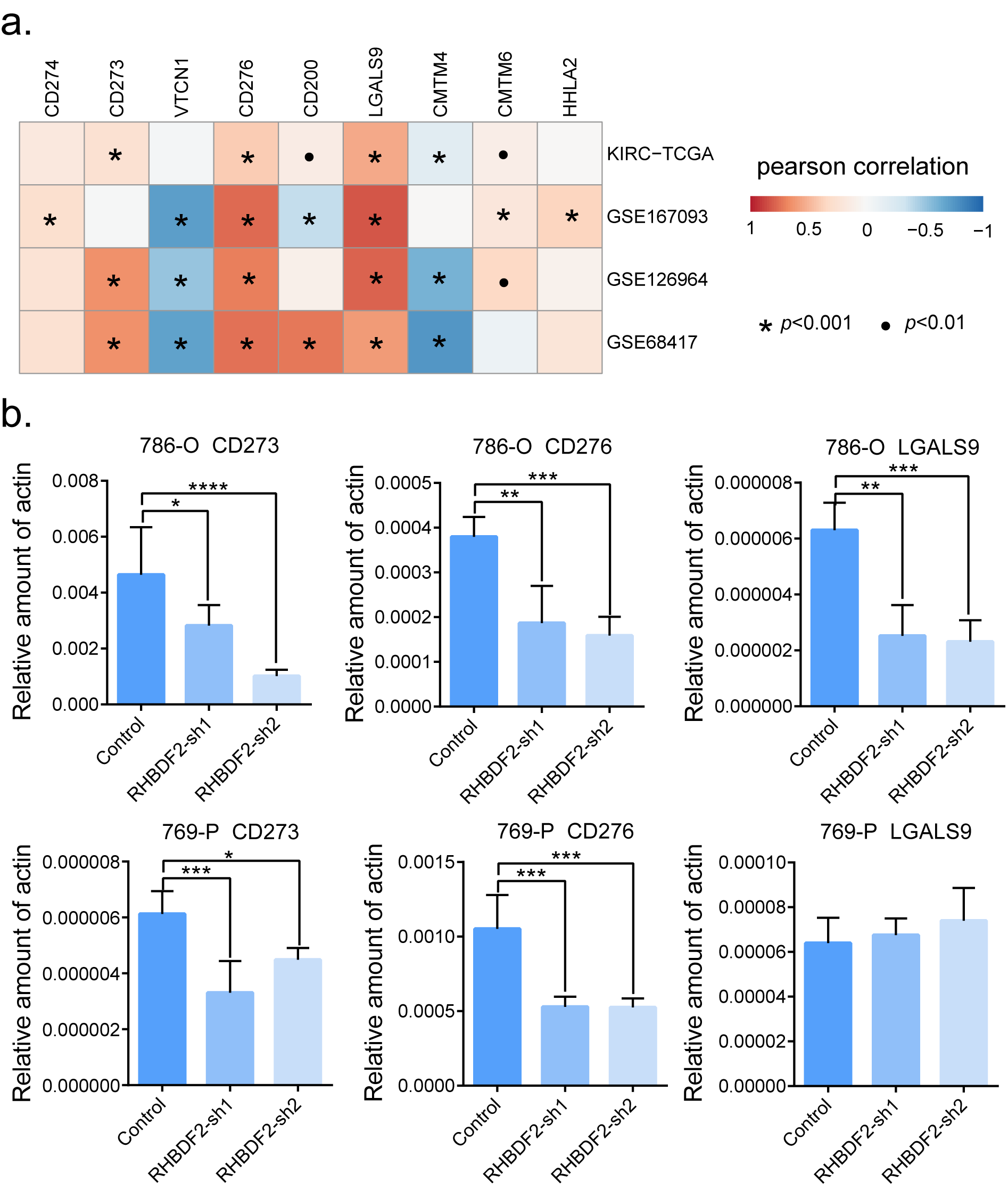

Supplement: Supplementary file 4 — Additional file 4: Fig. S3. Immune checkpoints analysis. (a) Pearson correlation of the expression of immune checkpoints and RHBDF2. (* p < 0.001, • p < 0.01). (b) Detection of the CD273, CD276 and LGALS9 expression in 786-O and 769-P with or without RHBDF2 knockdown (one-way ANOVA, * p < 0.05, ** p < 0.01, *** p < 0.005, **** p < 0.001). [file 12935_2021_2277_MOESM4_ESM.tif]

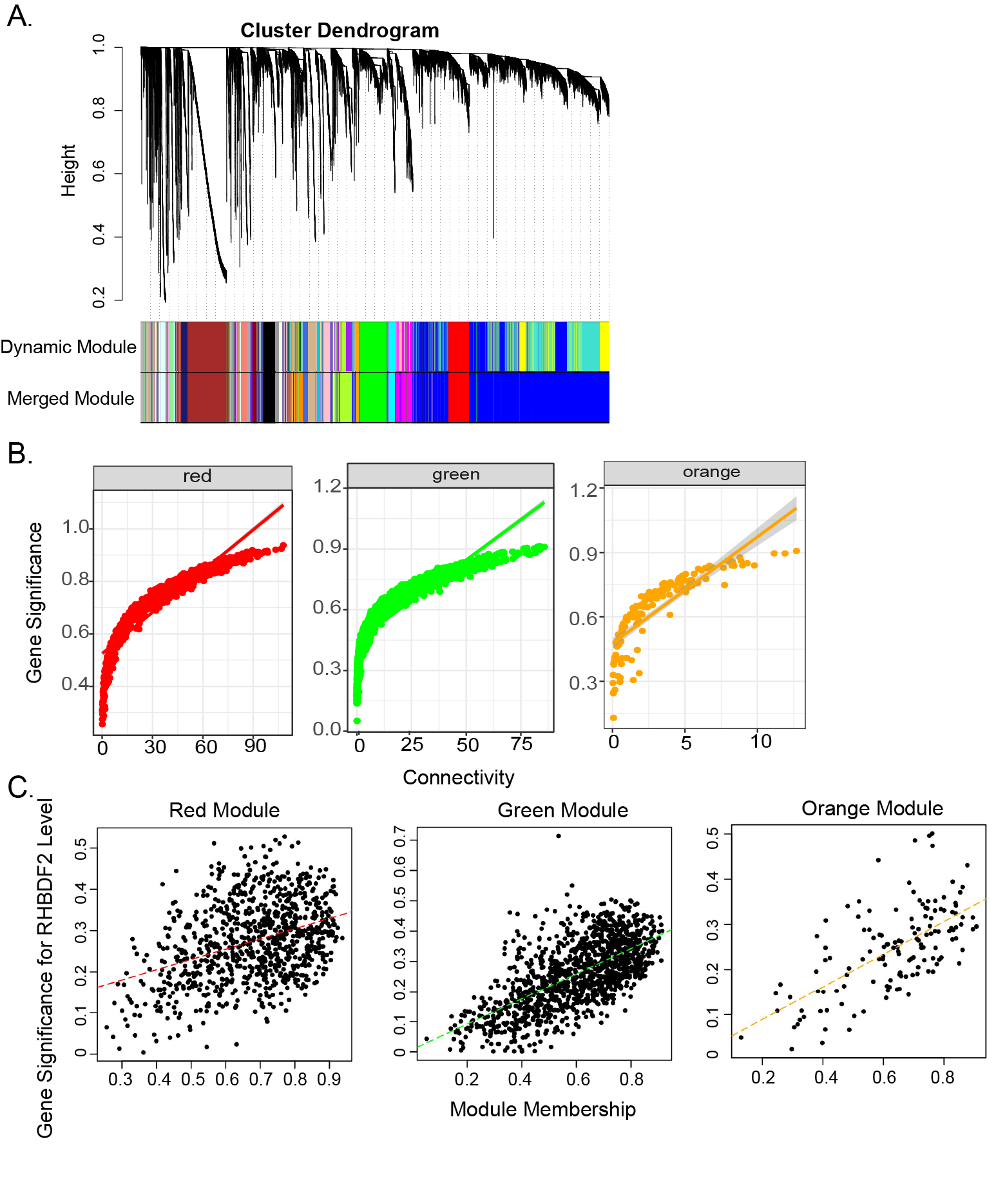

Supplement: Supplementary file 5 — Additional file 5: Fig. S4. Weighted Gene Correlation Network Analysis. (a) Co-expression modules for the KIRC transcriptome. (b) Module genes connectivity and genes significance in green, red and orange modules were present respectively. (c) Module genes significance for RHBDF2 expression and their module membership in green, red and orange modules were present. [file 12935_2021_2277_MOESM5_ESM.tif]

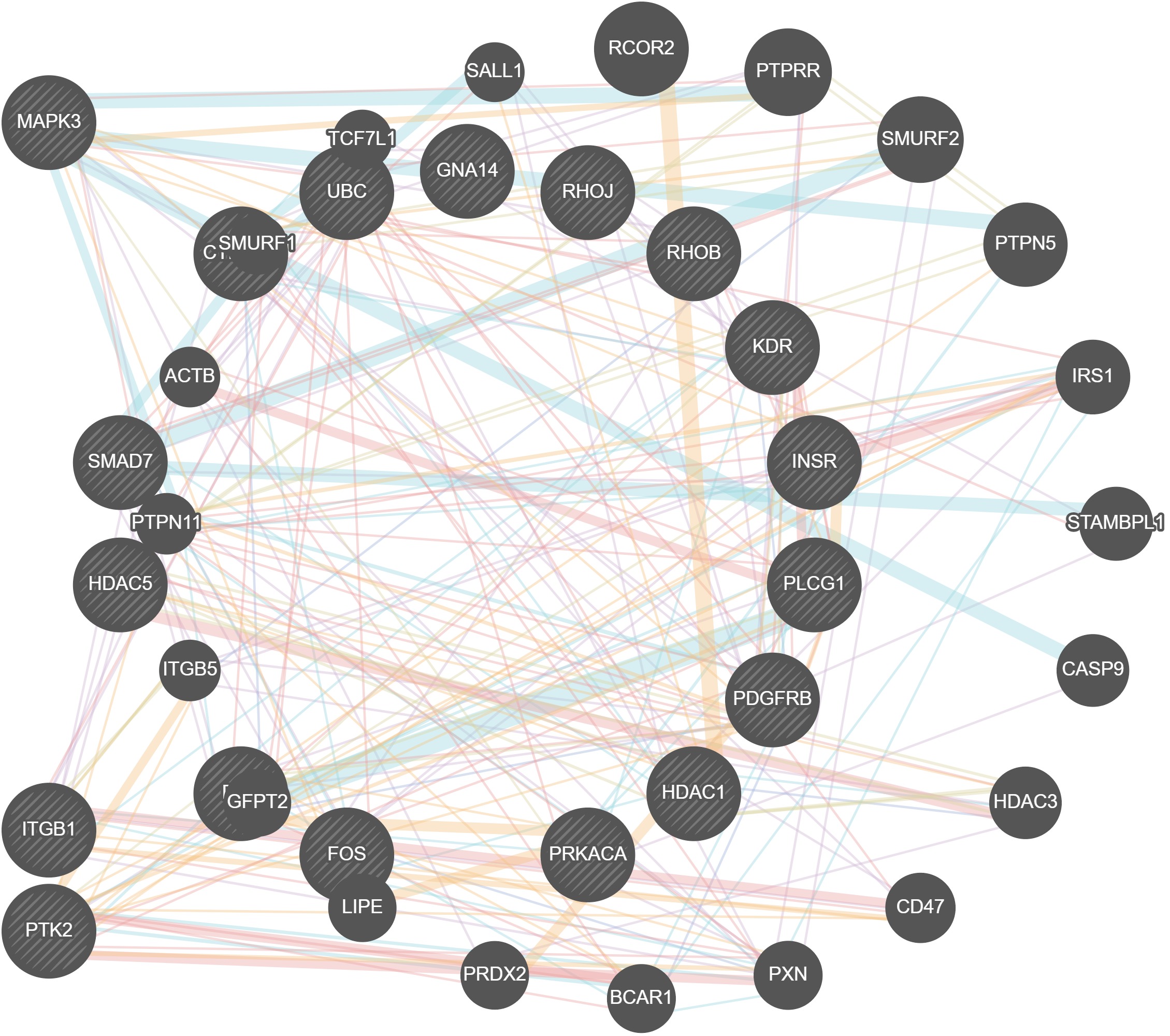

Supplement: Supplementary file 8 — Additional file 8: Fig. S5. Gene interactive network of hub genes in red module. [file 12935_2021_2277_MOESM8_ESM.jpg]

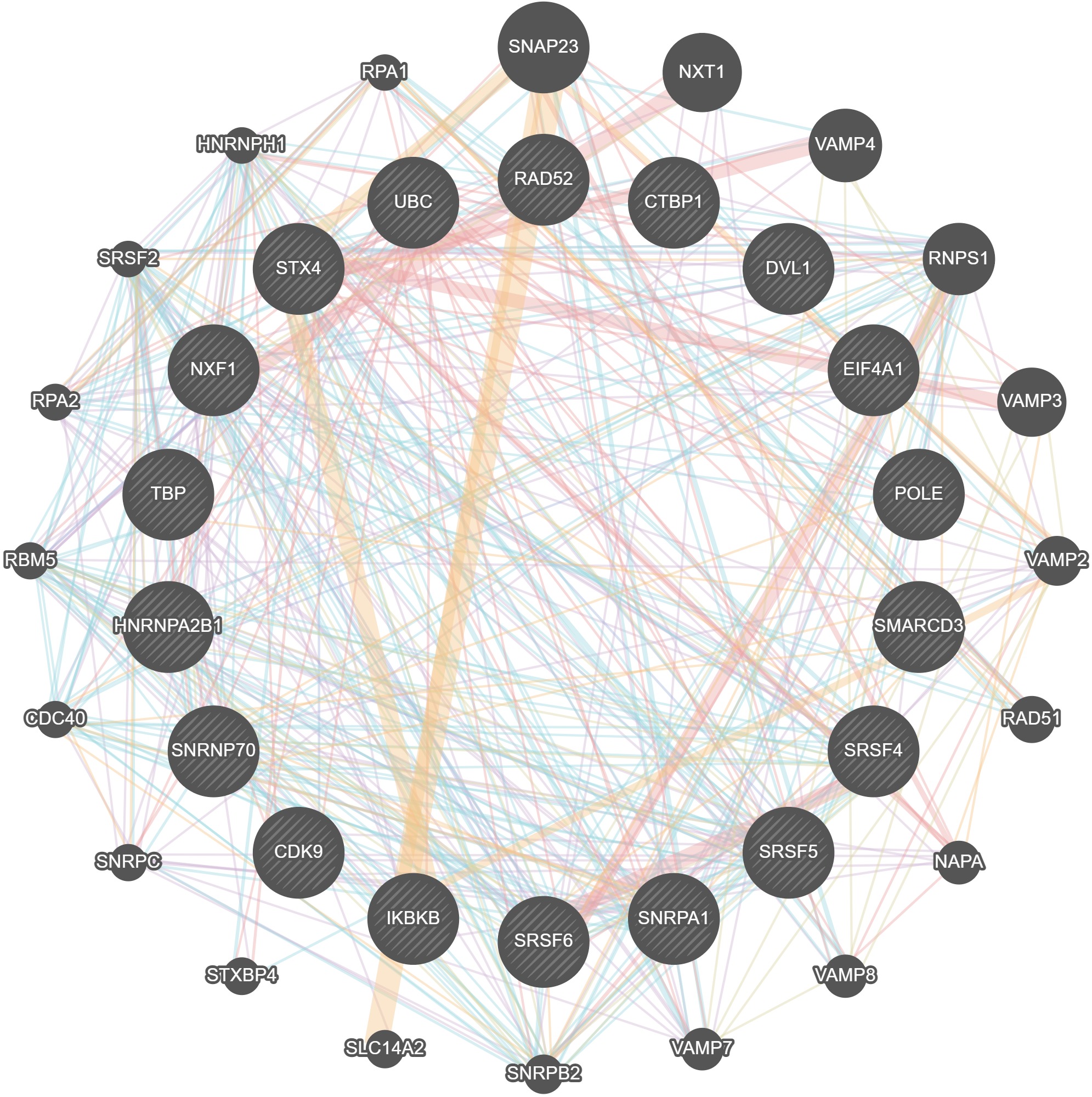

Supplement: Supplementary file 9 — Additional file 9: Fig. S6. Gene interactive network of hub genes in green module. [file 12935_2021_2277_MOESM9_ESM.jpg]

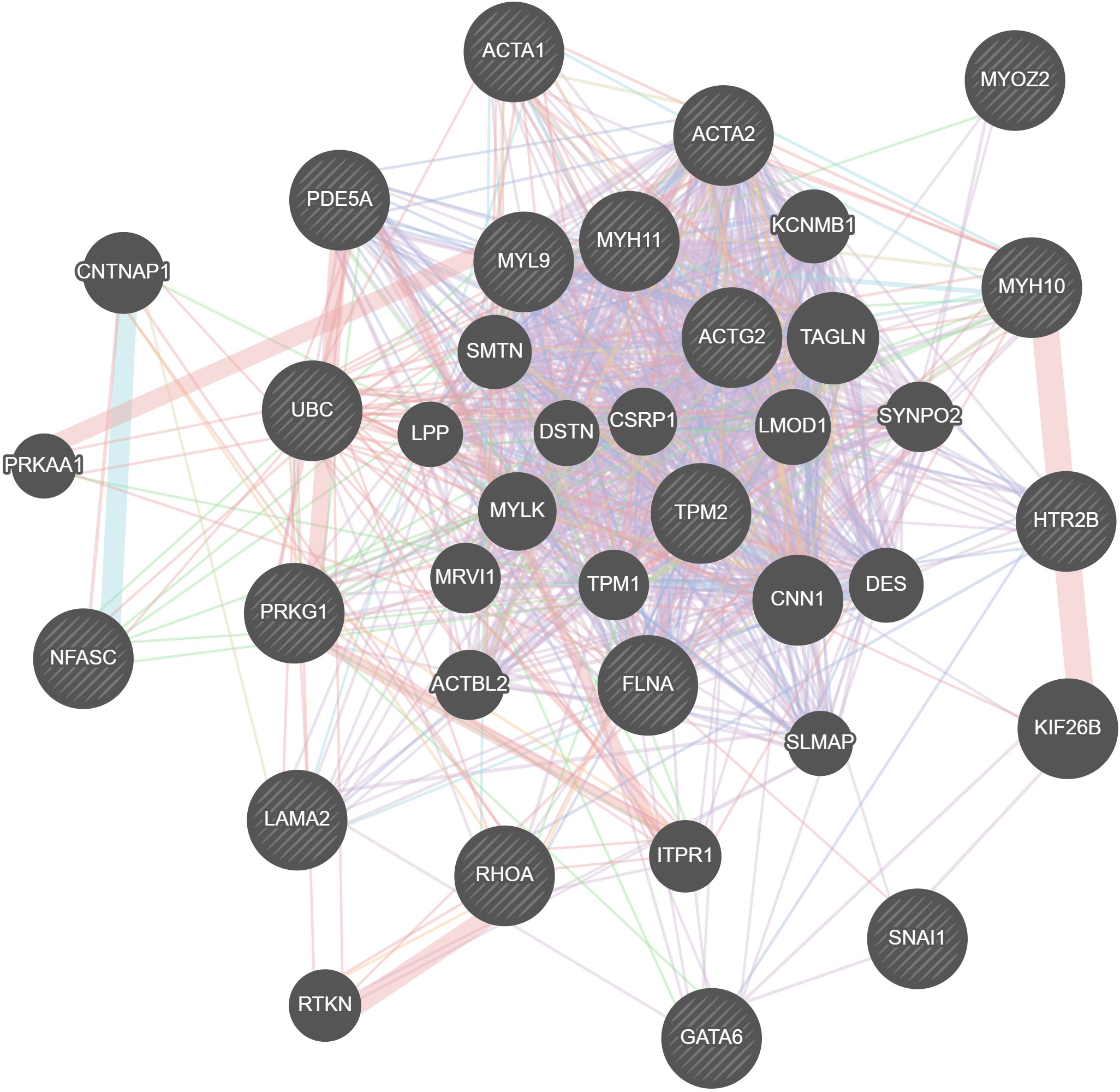

Supplement: Supplementary file 10 — Additional file 10: Fig. S7. Gene interactive network of hub genes in orange module. [file 12935_2021_2277_MOESM10_ESM.jpg]

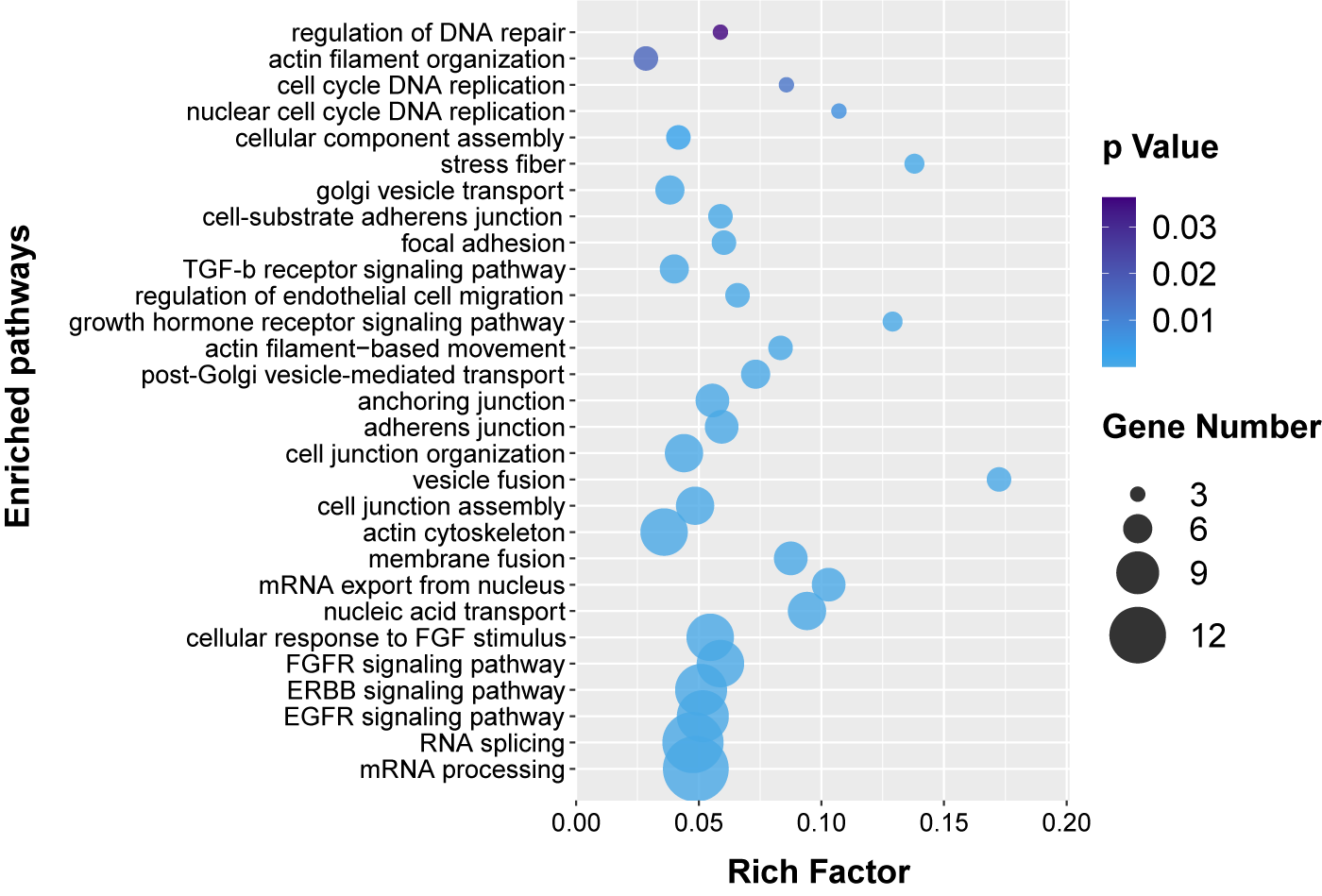

Supplement: Supplementary file 11 — Additional file 11: Fig. S8. Functional enrichment analyses of hub genes in red, green and orange modules. [file 12935_2021_2277_MOESM11_ESM.tif]

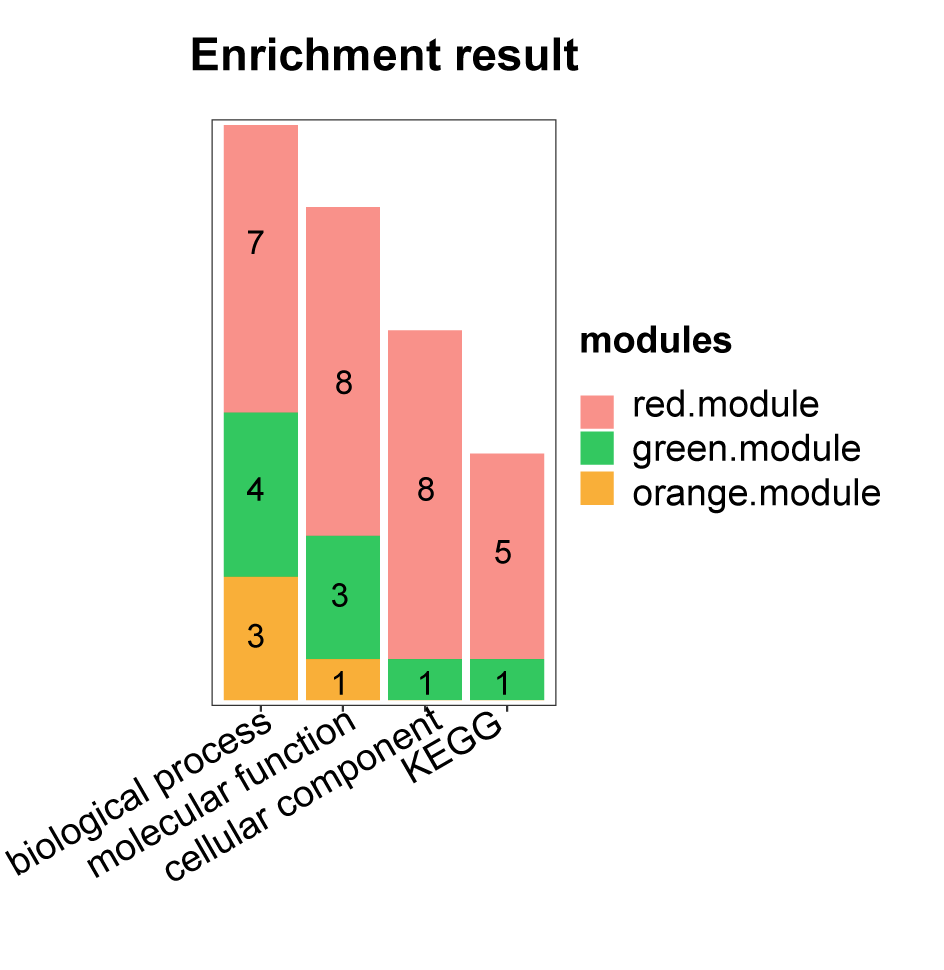

Supplement: Supplementary file 13 — Additional file 13: Fig. S9. Numbers of significant enrichment pathways and functions both in WGCNA and GSEA. [file 12935_2021_2277_MOESM13_ESM.tif]
